# Immune-related lincRNA pairs predict prognosis and therapeutic response in hepatocellular carcinoma

**DOI:** 10.1038/s41598-022-08225-w

**Published:** 2022-03-11

**Authors:** Yingna Zhang, Xiaofeng Yang, Lisha Zhou, Xiangting Gao, Xiangwei Wu, Xueling Chen, Jun Hou, Lianghai Wang

**Affiliations:** 1 NHC Key Laboratory of Prevention and Treatment of Central Asia High Incidence Diseases, The First Affiliated Hospital, Shihezi University School of Medicine, Shihezi, Xinjiang China; 2 Key Laboratory of Xinjiang Endemic and Ethnic Diseases, Shihezi University School of Medicine, Shihezi, Xinjiang China; 3 Department of Immunology, Shihezi University School of Medicine, Shihezi, Xinjiang China; 4 Department of Anatomy, Shihezi University School of Medicine, Shihezi, Xinjiang China; 5 Department of Pathology, The First Affiliated Hospital, Shihezi University School of Medicine, Shihezi, Xinjiang China

**Keywords:** Cancer, Biomarkers, Oncology

## Abstract

Growing evidence has demonstrated the functional relevance of long intergenic noncoding RNAs (lincRNAs) to tumorigenesis and immune response. However, immune-related lincRNAs and their value in predicting the clinical outcomes of patients with liver cancer remain largely unexplored. Herein, we utilized the strategy of iterative gene pairing to construct a tumor-specific immune-related lincRNA pairs signature (IRLPS), which did not require specific expression levels, as an indicator of patient outcomes. The 18-IRLPS we developed was associated with overall survival, tumor progression, and recurrence in liver cancer patients. Multivariate analysis revealed that the risk model was an independent predictive factor. A high IRLPS risk was correlated suppressive immune microenvironment, and IRLPS-high patients might benefit more from CD276 blockade or TMIGD2 agonist. Patients in the high-risk group were associated with elevated tumor mutation, increased sensitivity to dopamine receptor antagonists, cisplatin, doxorubicin, and mitomycin but more resistance to vinblastine. Mechanistically, IRLPS high scores might lead to poor prognosis by promoting cell proliferation and metabolic reprogramming. The prognostic significance of the 18-IRLPS was confirmed in independent cancer datasets. These findings highlighted the robust predictive performances of the 18-IRLPS for prognosis and personalized treatment.

## Introduction

Liver cancer is the sixth most commonly diagnosed cancer and the third leading cause of cancer death worldwide in 2020, while hepatocellular carcinoma (HCC) accounts for 70–85% of the total burden^1^. Surgical resection and liver transplantation are potentially curative treatment options available for early-stage HCC. However, 5-year survival rates following surgical resection remain relatively low for early-stage disease (17–53%), with recurrence rates as high as 70%^2,3^. At present, sorafenib and lenvantinib, the multi-targeted kinase inhibitors, are used as first-line therapy for advanced HCC, remaining unsatisfactory in the clinical practice^4^. Moreover, HCC is a highly heterogeneous tumor, which considerably cuts down the efficacy of clinical treatments and makes the survival prediction quite complicated^5^. The efficacy and safety of nivolumab, an immunotherapy targeting PD-1, have been explored in patients with HCC. However, only ~ 20% of participants respond to the treatment^6,7^. Several biomarkers, including PD-L1 expression, microsatellite instability, and tumor mutational burden (TMB), have been approved for selecting patients with other malignancies who will benefit from the immune-checkpoint blockade therapy^8^. However, there are few robust predictive biomarkers available in HCC, with the use of PD-L1 expression being of limited value^6,7^. Thus, the perusal of the tumor microenvironment and identifying novel and promising biomarkers have become imperative for improved treatment in HCC patients.

Long noncoding RNAs (lncRNAs), which are RNA transcripts longer than 200 bp and unable to code proteins, could regulate gene expression by interacting with DNA, RNA, and proteins to exhibit either enhancement or inhibition^9,10^. Increasing evidence support that lncRNAs play critical roles in tumorigenesis and progression of HCC^11,12^. Moreover, lncRNAs are reported as crucial regulators of cancer immunity, such as antigen release and immune activation, which contribute to the malignant phenotypes of cancer^13–15^. There is growing evidence that immune-related lncRNAs may be novel disease biomolecules for clinical cancer treatment and possess valuable prognostic significance for survival^16,17^. However, batch effects on the detected gene expression profiles due to different platforms and testing time may lead to inaccuracy of prognostic prediction using gene signatures according to their exact expression levels^18^.

Recently, a novel algorithm for normalizing and scaling the expression matrix based on the relative ranking of gene expression levels has been proposed to eliminate the potential defects mentioned above^19,20^. In this study, we were inspired by the strategy of gene pairing and aimed to discover an immune-related intergenic lncRNAs (lincRNAs)-based risk model for predicting clinically relevant outcomes in patients with HCC. By iteratively comparing the relative expression of tumor-specific immune-associated lincRNA pairs in each sample, we developed a valid signature with no requirement of specific expression levels. We estimated its predictive value among patients with HCC for prognostic effectiveness, tumor immune infiltration, and therapeutic liability in immunotherapy, targeted therapy, and chemotherapy. We also found that the signature of lincRNA pairs was associated with enhanced cell cycle and altered metabolism. Lastly, we validated its predictive efficacy in multiple cancer types.

## Results

### Establishment of immune-related lincRNA pairs signature with prognostic significance

**Figure 1:**
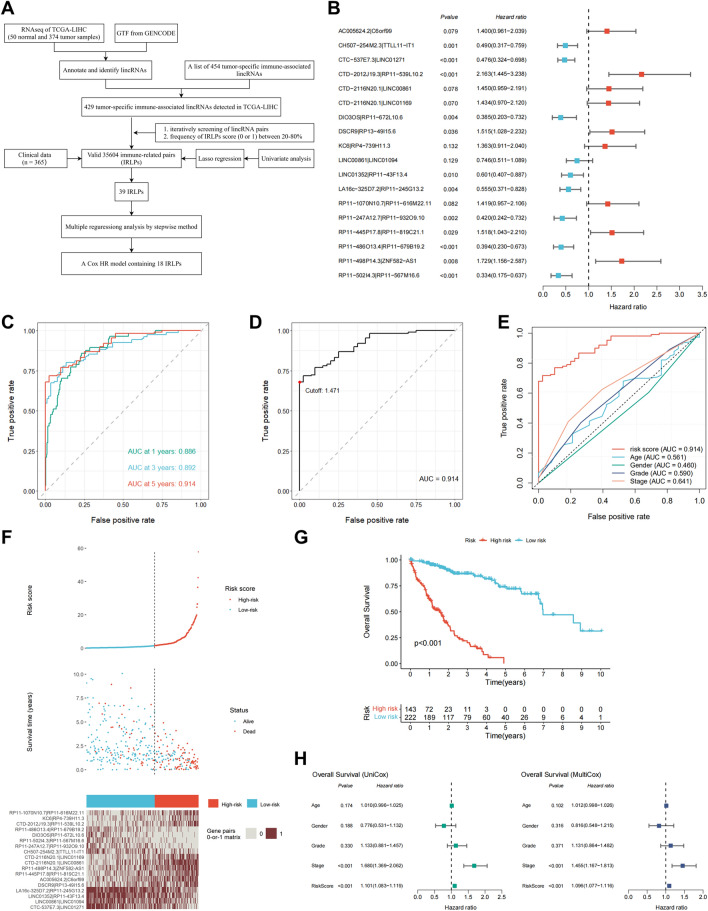
Construction of immune-related lincRNA pairs signature with prognostic significance. (**A**) Flow Chart of identifying tumor-specific immune-related lincRNA pairs signature (IRLPS). (**B**) Forest map showing the 18 IRLPS identified by multivariate Cox proportional hazard regression analysis of overall survival. (**C**) The 1-, 3-, and 5-year ROC curves of the optimal model for overall survival. (**D**) The optimal cutoff point for the IRLPS on the 5-year ROC curve. (**E**) Comparison of 5-year ROC curves of the IRLPS with that of common clinical characteristics. (**F**) The distribution of the risk score, patients’ survival status, and IRLPs expression pattern. (**G**) Kaplan–Meier survival curves of overall survival between patients with higher and lower risk scores of the IRLPS. (**H**) Univariate (left) and multivariate (right) Cox hazard ratio analysis of overall survival.

The strategy for identifying tumor-specific immune-related lincRNA pairs signature (IRLPS) in this study is shown in Fig. 1A. First, we selected 454 lincRNAs, which were potential tumor-intrinsic (highly expressed in tumor tissues compared with adjacent normal tissues but not expressed in immune tissues) immune regulators from a previous study^21^. Next, we retrieved the transcriptome profiles of The Cancer Genome Atlas Liver Hepatocellular Carcinoma (TCGA-LIHC) cohort and separated the data of lncRNA and mRNA using the GENCODE annotation file. Among the 454 tumor-specific immune-associated lincRNAs, 429 lincRNAs were detected in the TCGA-LIHC dataset and selected for subsequent analysis (Supplementary Table 1). Using an iterative 0-or-1 matrix screening, we identified 35,604 valid lincRNA pairs. After a univariate analysis followed by a modified Lasso penalized regression, 39 pairs were extracted. At last, 18 lincRNA pairs were included in the IRLPS using a stepwise method with a multivariate Cox proportional hazards model for overall survival (Fig. 1B). To assess the prognostic performance of the risk model, the areas under curve (AUC) for each ROC curve of the 18-IRLPS was calculated. The AUC values ranged from 0.886 to 0.914 at the 1-, 3-, and 5-year ROC curves for overall survival, confirming the optimality of the IRLPS (Fig. 1C). Next, we determined the maximum inflection point (1.471) as the optimal cutoff value for an ideal IRLPS on the 5-year receiver operating characteristic (ROC) curve using the Akaike information criterion (Fig. 1D). We also compared the 5-year ROC curve of the IRLPS with common clinical characteristics, showing the superiority of the risk model (Fig. 1E). Based on the cutoff value determined above, 365 acceptable patients from the TCGA-LIHC cohort were stratified into the high- and low-risk groups. The distribution of the IRLPS risk score, survival status, and IRLPs expression pattern was revealed in Fig. 1F. Patients in the high-risk group exhibited an inferior clinical outcome compared with those in the low-risk group. Kaplan–Meier analysis showed that patients in the high-risk group had significantly shorter overall survival time than those in the low-risk group (Fig. 1G). Moreover, multivariate Cox regression analysis indicated that the prognostic performance of the risk score was independent of other clinical factors for overall survival prediction after being adjusted by other clinical characteristics, including age, gender, grade, and stage (Fig. 1H). We also investigated the possible correlations between the risk score and clinicopathological features. The alluvial diagram, pie chart, and strip chart showed that the high-risk group was significantly corresponded to more patients being dead and with advanced clinical stage, M stage, and T stage (Supplementary Fig. 1A–C). On the other hand, patients with higher T stage, clinical stage, and tumor grade were significantly associated with increased risk scores (Supplementary Fig. 1D–F).

To further examine whether the IRLPS is related to tumor progression and recurrence, we used progression-free survival and disease-free interval to investigate the effectiveness of the IRLPS for risk prediction. The AUC values for predicting patients' progression status by the IRLPS were 0.727, 0.704, and 0.770 at the 1-, 3-, and 5-year ROC curves and achieved better predictive performance than other clinical characteristics (Supplementary Fig. 2A,B). Kaplan–Meier analysis demonstrated that patients in the high-risk group significantly correlated with unfavorable progression-free survival (Supplementary Fig. 2C). We also performed multivariate Cox regression analysis of progression-free survival and found that the IRLPS remained an independent predictor for progression-free survival (HR = 1.050, 95% CI 1.027–1.074; Supplementary Fig. 2C). The IRLPS also exhibited the superiority for predicting tumor recurrence, with the AUC values being 0.757, 0.694, and 0.797 at the 1-, 3-, and 5-year ROC curves (Supplementary Fig. 2E,F). Patients in the high-risk group experienced significantly reduced disease-free interval than those in the low-risk group (Supplementary Fig. 2G). Multivariate Cox regression analysis of disease-free interval indicated that increasing IRLPS risk score was independently associated with a greater probability of disease recurrence (HR = 1.056, 95% CI 1.025–1.088; Supplementary Fig. 2H).

### Correlation between the risk model and tumor immune microenvironment

**Figure 2:**
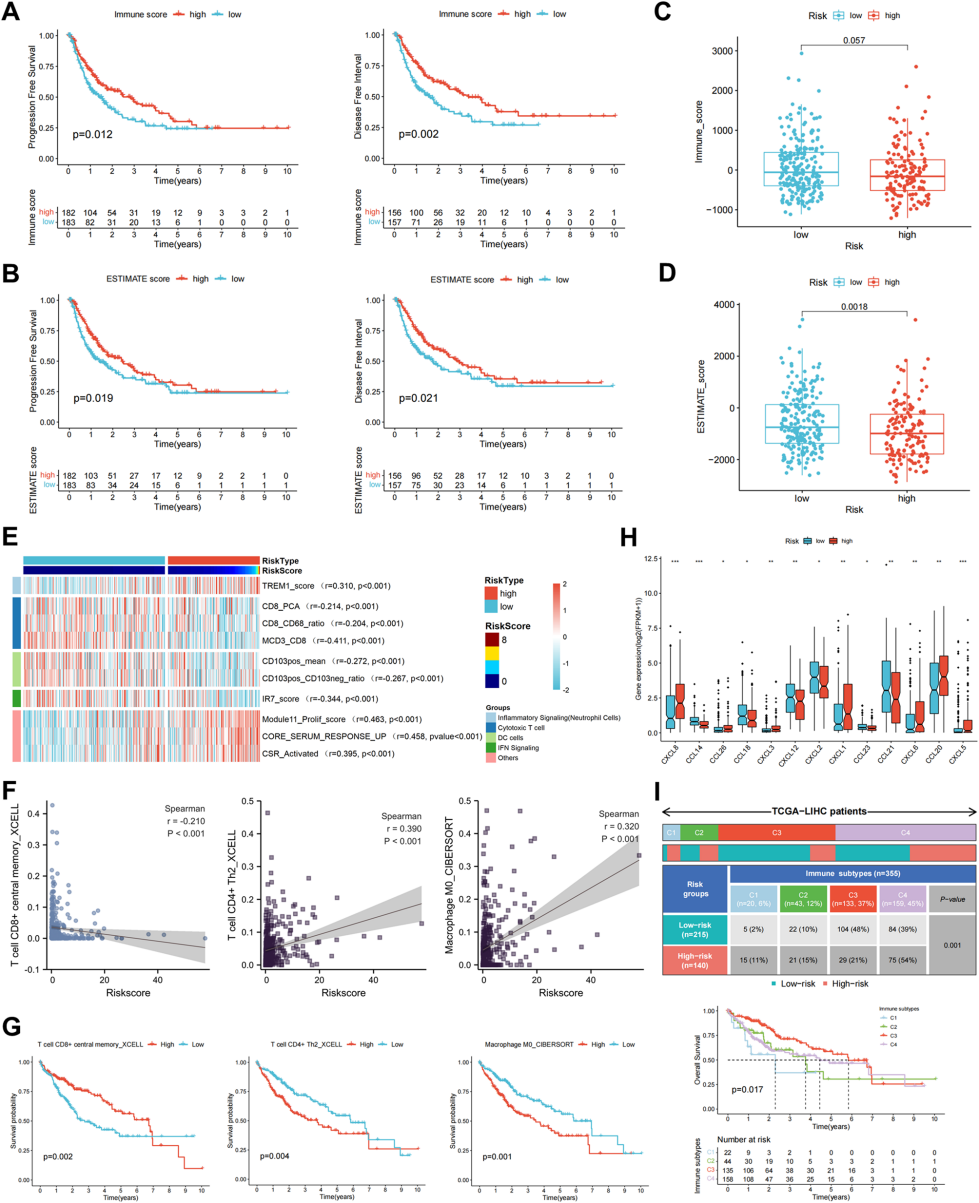
Correlation between the prognostic risk score with tumor immune microenvironment. (**A**, **B**) Low immune score (**A**) or ESTIMATE score (**B**) was associated with shortened progression-free survival (left) and disease-free interval (right) in TCGA-LIHC patients. (**C**, **D**) Box plot comparing the immune score (**C**) or ESTIMATE score (**D**) between the high- and low-risk groups. (**E**) Correlation heatmap of the IRLPS risk score and enrichment scores of representative immune-related signatures. (**F**) Association between the risk score of IRLPS and tumor-infiltrating immune cells including central memory CD8^+^ T cells, Th2 CD4^+^ T cells, and M0 macrophages. (**G**) Kaplan–Meier survival curves of overall survival between patients with high- and low-abundance of tumor-infiltrating central memory CD8^+^ T cells, Th2 CD4^+^ T cells, and M0 macrophages. (**H**) Box plot comparing gene expression of chemokines between the high- and low-risk groups by Wilcoxon signed-rank test. (**I**) Heatmap and table showing the distribution of immune subtypes between the IRLPS risk groups, compared using the chi-square test (top). Kaplan–Meier survival curves of overall survival between patients within the indicated immune subtypes (bottom).

We subsequently investigated whether the IRLPS was related to the tumor immune microenvironment. Kaplan–Meier survival curves revealed that patients with low immune scores (representing the infiltration of immune cells in tumor tissue) or ESTIMATE scores (inferring tumor purity) from the TCGA-LIHC cohort had a worse progression-free survival and disease-free interval (Fig. 2A,B). Intriguingly, the immune scores of samples within the IRLPS-high group showed a declining trend, whereas the ESTIMATE scores were significantly lower in tumors with higher risk scores (Fig. 2C,D). We applied Spearman’s correlation analysis to the IRLPS risk score and the enrichment scores of 68 immune signatures calculated using the single-sample gene set enrichment analysis (ssGSEA)^21^. Results showed that the risk score was positively associated with neutrophil cells but inversely correlated with cytotoxic T cell, DC cells, and IFN signaling (Fig. 2E). We also quantified the enrichment of a set of marker genes for tumor-infiltrating immune cells to assess immune functions^22^. Consistently, the enrichment scores of cytolytic activity, T cell co-stimulation, HLA, type I IFN response, type II IFN response, and inflammation-promoting were significantly lower in the high-risk group (Supplementary Fig. 3A). We further explored the potential correlation between the risk score of the IRLPS and immune cell infiltration abundance calculated by seven commonly acknowledged deconvolution methods, including TIMER, XCELL, QUANTISEQ, MCPcounter, EPIC, CIBERSORT, and CIBERSORT-ABS^23^. Results showed a higher risk score was negatively associated with central memory CD8^+^ T cells while positively related to Th2 CD4^+^ T cells and M0 macrophages (Fig. 2F, Supplementary Fig. 3B,C). Notably, patients with a low infiltration level of central memory CD8^+^ T cells or high abundance of Th2 CD4^+^ T cells and M0 macrophages exhibited significantly reduced overall survival probability (Fig. 2G, Supplementary Fig. 3D). Additionally, we investigated the relationship between the risk score of IRLPS and chemokine expression levels using the TISIDB database^24^. Elevated expression of CXCL1, CXCL3, CXCL5, CXCL6, CXCL8, CCL20, and CCL26 was observed in the high-risk group while CCL14, CCL18, CCL21, CCL23, CXCL2, CXCL12, and CXCL23 were declined (Fig. 2H). Immune subtypes spanning multiple cancer types have been proposed to define immune response patterns affecting patient prognosis. Subtype C1 (wound healing) had a high proliferation rate and a Th2 cell bias. In contrast, the C3 (inflammatory) subtype is characterized by low to moderate tumor cell proliferation and lower levels of somatic copy number alterations than the other subtypes^25^. For liver cancer, patients within the C3 subtype displayed the most favorable prognosis while C1 the worst. Moreover, there were more C1 subtypes and fewer C3 subtypes in the IRLPS-high group than the low-risk group (Fig. 2I). On the other hand, C3 subtype constituent tumors showed remarkably lower risk scores of the IRLPS than C1 (Supplementary Fig. 3E).

### Correlation of the IRLPS to immune checkpoint-related genes and somatic alteration

**Figure 3:**
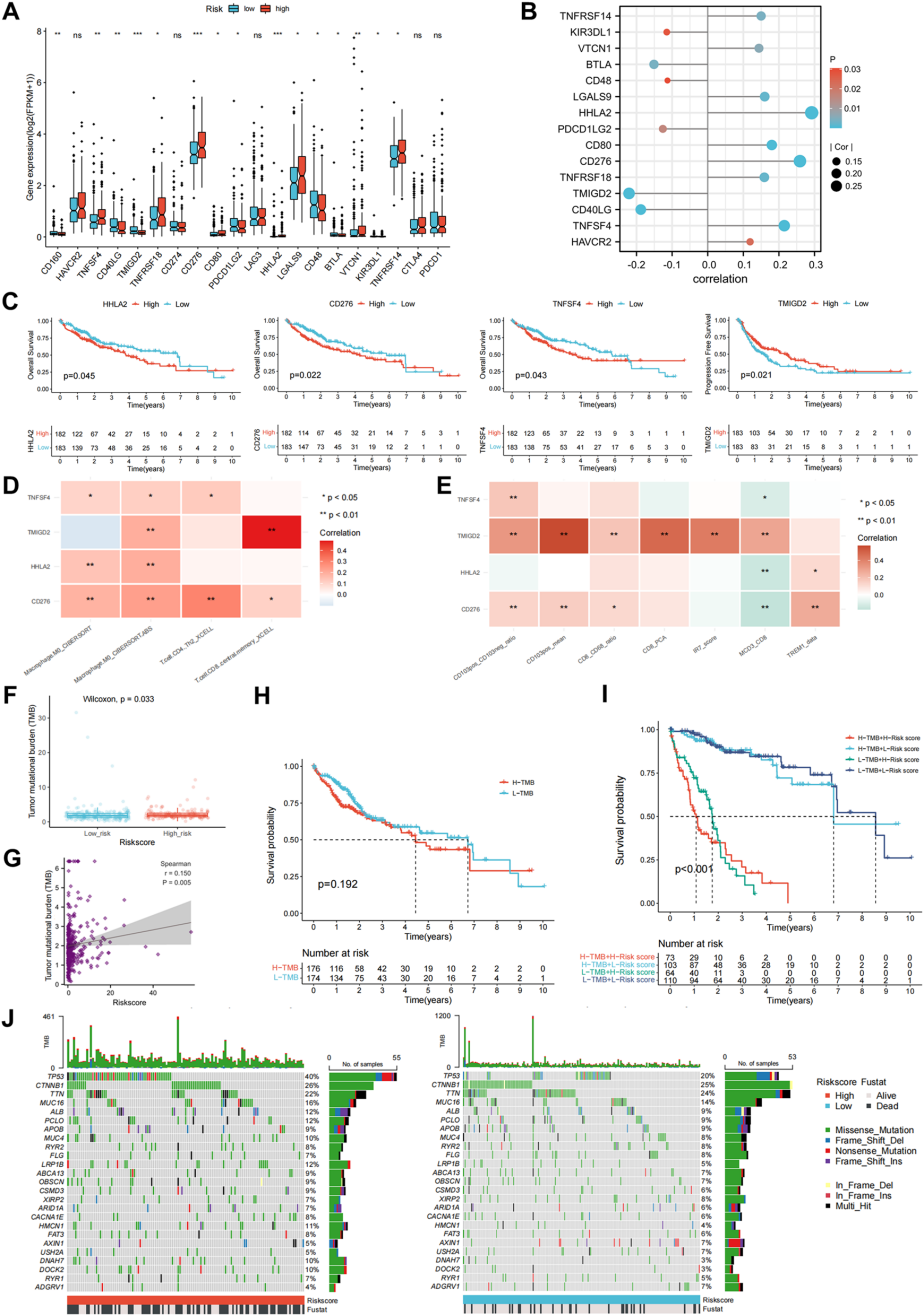
Correlation of the IRLPS to immune checkpoint-related genes and somatic alteration. (**A**) Expression of 20 immune checkpoint-related genes in high- and low-risk groups. (**B**) Lollipop diagram showing the Spearman's correlation between the indicated immune genes with the risk score of IRLPS. (**C**) Kaplan–Meier survival curves between patients with the high and low expression level of HHLA2, CD276, TNFSF4, and TMIGD2. (**D**) Correlation matrix of the indicated immune gene expression and tumor-infiltrating M0 macrophages, Th2 CD4^+^ T cells, and central memory CD8^+^ T cells. (**E**) Correlation matrix of the indicated immune gene expression and enrichment scores of representative immune-related signatures. (**F**) Comparison of tumor mutational burden (TMB) between the low- and high-risk groups in the TCGA-LIHC cohort. (**G**) Scatterplot depicting a positive correlation between risk score and mutation load. (**H**) Kaplan–Meier survival curves for patients with high and low TMB, dividing with the median TMB as a cutoff. (**I**) Kaplan–Meier survival curves of overall survival among four patient groups stratified by TMB and the risk score of IRLPS. (**J**) The oncoPrint of tumors with high (left) and low (right) risk scores. Individual patients are represented in each column.

To further explore the relationship between the IRLPS and tumor immunity, we assessed the risk score and immunotherapy-relevant biomarkers correlation. Immune checkpoint-related genes could trigger the immunosuppressive tumor environment and are reported as predictive biomarkers for immunotherapy in multiple malignancies. Gene expression levels of TNFSF4, TNFRSF18, CD276, CD80, HHLA2, LGALS9, VTCN1, and TNFRSF14, but not HAVCR2, LAG3, CTLA4, and PDCD1, were significantly upregulated in IRLPS-high patients (Fig. 3A). Moreover, we found that the risk score was positively correlated with HHLA2 (r = 0.292), CD276 (r = 0.259), TNFSF4 (r = 0.215) and inversely related to TMIGD2 (r = − 0.221; Fig. 3B). Notably, Kaplan–Meier analysis showed that the higher expression of HHLA2, CD276, and TNFSF4 was significantly associated with inferior prognosis in overall survival while patients with lower TMIGD2 levels had worse progression-free survival (Fig. 3C). We further analyzed their correlation with the IRLPS-associated tumor-infiltrating immune cells and immune signatures (Fig. 2E,F). Results indicated that CD276 was positively correlated with the abundance of Th2 CD4^+^ T cells (r = 0.301) and M0 macrophages (r = 0.236) while TMIGD2 was related to enriched central memory CD8^+^ T cells (r = 0.487; Fig. 3D). We also observed an association of CD276 with neutrophil cells (TREM1_data; r = 0.269) and TMIGD2 with DC cells (CD103pos_mean, r = 0.569; CD103pos_CD103neg_ratio, r = 0.332), cytotoxic T cell (CD8_PCA, r = 0.479; MCD3_CD8, r = 0.287), and IFN Signaling (IR7_scorer, r = 0.423; Fig. 3E). These results indicated that patients in the IRLPS-high group might benefit more from CD276 blockade or TMIGD2 agonist.

Given the clinical implications of TMB, it is worth exploring its correlation with the risk score of IRLPS. TMB was significantly higher in patients from the high-risk group than those from the low-risk group and positively correlated with the risk score (Fig. 3F,G). However, no significant difference in overall survival was observed between patients with high and low TMB (Fig. 3H). We next evaluated the synergistic effect of the IRLPS and TMB in prognostic prediction. Stratified survival analysis of the four patient groups revealed that the TMB status did not interfere with IRLPS and the IRLPS subgroups remained significant survival differences in both high and low TMB subgroups (Fig. 3I), suggesting that the IRLPS could serve as a predictive indicator independent of TMB. Furthermore, we compared the distribution of somatic varients in the top 25 driver genes with the highest alteration frequency between the high- and low-risk groups (Fig. 3J). Results showed a significantly greater alteration frequency of TP53, DOCK2, DNAH7, HMCN1, and LRP1B in the high-risk groups (Supplementary Table 2), which provided potential directions for interpreting the underlying mechanisms of the IRLPS.

### Candidate compounds/chemotherapies targeting the IRLPS

**Figure 4:**
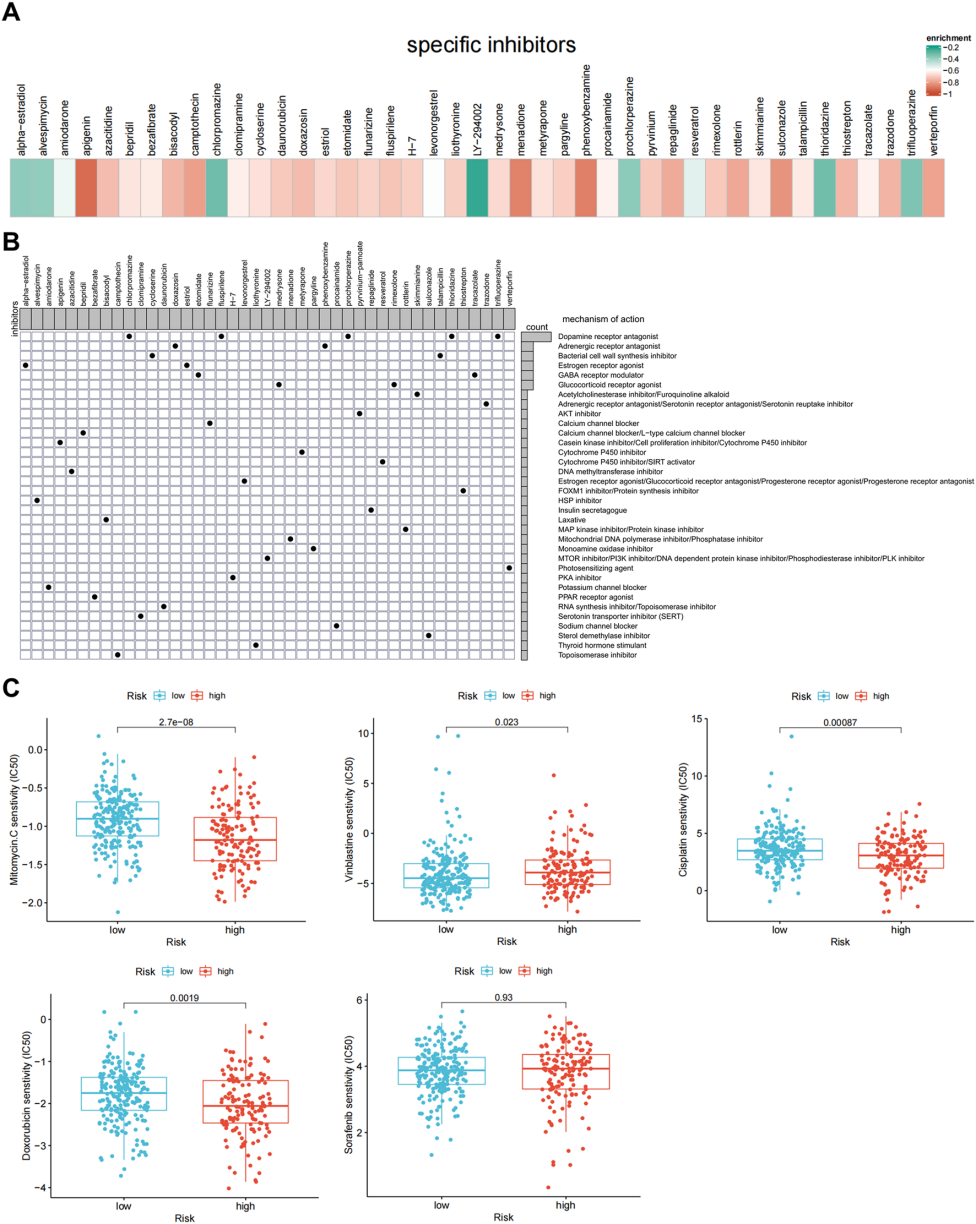
Small molecular compounds identification and sensitivity prediction for chemotherapies. (**A**) Heatmap showing the enrichment score of candidate compounds from the CMap. (**B**) Heatmap showing each compound from the CMap that shares mechanisms of action (rows), sorted by descending number of compounds that share mechanisms of action. (**C**) Sensitivity of the indicated drugs in the two risk groups, compared by Wilcoxon signed-rank test.

To understand the potential therapeutic value of the IRLPS in drug sensitivity prediction, we first employed the Connectivity Map (CMap) to discover candidate small molecular compounds that might target the IRLPS-associated pathways. A total of 43 compounds were significantly enriched (Fig. 4A). CMap mode-of-action (MoA) analysis revealed 34 mechanisms of action shared by these compounds (Fig. 4B). Five compounds (chlorpromazine, fluspirilene, prochlorperazine, thioridazine, and trifluoperazine) shared the MoA of dopamine receptor antagonist, which has been reported to inhibit stemness-related tumorigenicity^26,27^. We also identified shared MoA of adrenergic receptor antagonist (doxazosin and phenoxybenzamine), estrogen receptor agonist (alpha-estradiol and estriol), GABA receptor modulator (etomidate and tracazolate), glucocorticoid receptor agonist (medrysone and rimexolone), and the enrichment of the cell proliferation inhibitor apigenin.

We further explored the role of IRLPS in the sensitivity prediction for common administrating chemotherapeutic drugs, including doxorubicin, cisplatin, mitomycin, vinblastine, and sorafenib. Patients in the high-risk group showed increased sensitivity to cisplatin, doxorubicin, and mitomycin but more resistance to vinblastine. However, no significant difference for sorafenib was observed between the two risk groups (Fig. 4C). Together, these results imply that the IRLPS may be a potential biomarker for identifying patients who are more likely to benefit from a tailored treatment strategy.

### Pathway and gene network features associated with the IRLPS

**Figure 5:**
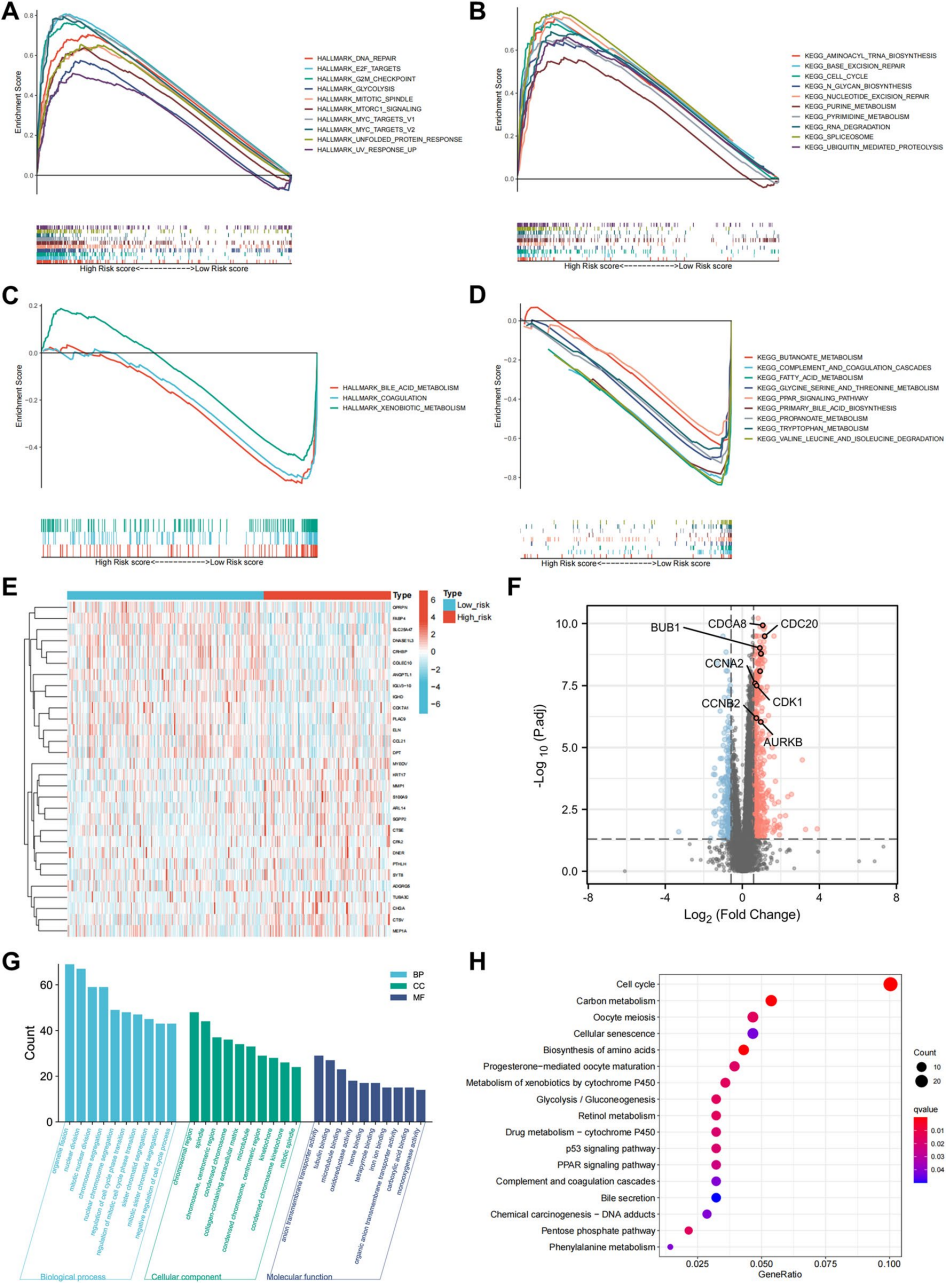
Pathway and gene network features associated with the IRLPS. (**A**, **B**) Top ten Hallmark (**A**) and KEGG (**B**) pathways significantly associated with the high-risk group in TCGA-LIHC. (**C**, **D**) The significantly enriched Hallmark (**C**) and KEGG (**D**) pathways in the low-risk group. (**E**) Heatmap of the differentially expressed genes. (**F**) Volcano plot of significant differentially expressed genes between the two risk groups. Significantly upregulated and downregulated genes in the high-risk group are represented as red and blue dots, respectively. The top ten hub genes are labeled. (**G**) GO enrichment analyses of the differentially expressed genes. (**H**) KEGG pathway enrichment analyses of the differentially expressed genes.

To identify the potential biological processes related to the IRLPS, GSEA was employed to assess the Hallmark and KEGG pathways. We found that the high-risk group was significantly associated with carcinogenic pathways such as cell cycle (E2F targets, G2M checkpoint, mitotic spindle, MYC targets), DNA repair, and glycolysis (Fig. 5A,B). In contrast, bile acid metabolism and amino acid metabolism were enriched in the low-risk group (Fig. 5C,D). We further identified 481 significantly upregulated and 190 downregulated genes in the high-risk group compared with the low-risk group (Fig. 5E,F). Enrichment analysis was performed to illustrate the functional annotations of these differentially expressed genes. We observed the enrichment of cell division- and cell cycle regulation-related GO terms (Fig. 5G), as well as KEGG cell cycle and carbon metabolism pathways (Fig. 5H). Collectively, these findings suggested that the IRLPS might lead to poor prognosis by promoting cell proliferation and metabolic reprogramming.

**Figure 6:**
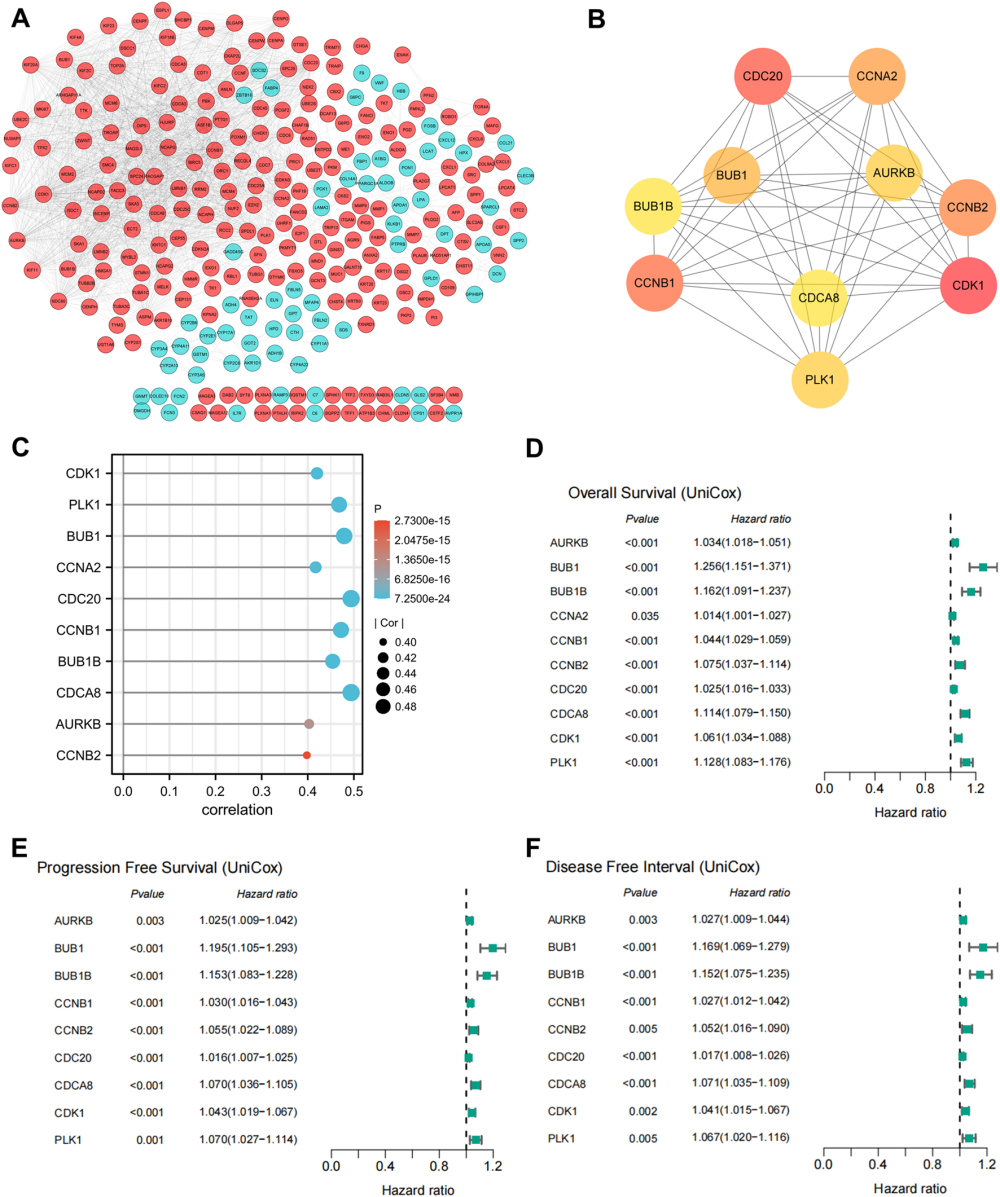
Identification of hub genes involved in the IRLPS. (**A**) A PPI network of the differentially expressed genes with the STRING confidence score > 0.9. Thicker edges between nodes indicate stronger combined scores. (**B**) Identification of the top ten hub genes in the PPI network using the multi-network clustering (MNC) algorithm. Red nodes represent genes with high MNC scores, while yellow nodes represent genes with lower MNC scores. (**C**) Lollipop diagram showing the Spearman's correlation between the ten hub genes with the IRLPS risk score. (**D**–**F**) Forest plot of univariate Cox regression analyses for overall survival (**D**), progression-free survival (**E**), and disease-free interval (**F**) in TCGA-LIHC, showing hub genes with significance.

To further narrow the scope, a protein–protein interaction network (containing 284 nodes and 1827 edges) was constructed (Fig. 6A). Then the top ten hub genes, including CDK1, CDC20, CCNB1, CCNB2, BUB1, AURKB, PLK1, BUB1B, CDCA8, and CCNA2, were identified (Fig. 6B). Their expression was positively correlated with the IRLPS risk score (r > 0.3; Fig. 6C). Nine of the ten hub genes, except CCNA2, were significantly associated with increased hazard ratios for overall survival, progression-free survival, and disease-free interval based on Cox regression analysis (Fig. 6D–F).

### Confirmation of the IRLPS in independent cancer datasets

**Figure 7:**
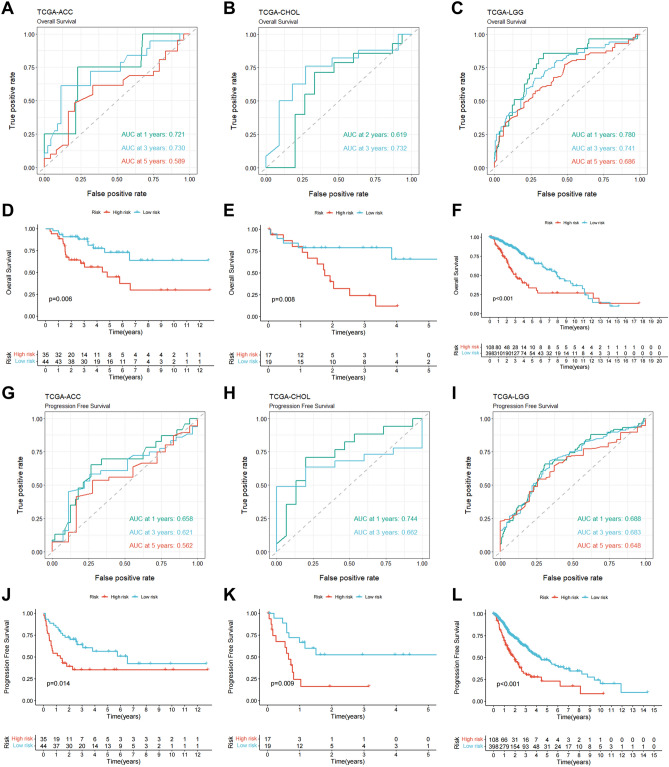
Prognostic significance validation of the 18-IRLPS in independent cohorts. (**A**–**C**) The time-dependent ROC curves of the IRLPS for predicting overall survival in TCGA-ACC (**A**), TCGA-CHOL (**B**), and TCGA-LGG (**C**). (**D**–**F**) Kaplan–Meier survival curves of overall survival between patients with higher and lower risk scores of the IRLPS in TCGA-ACC (**D**; n = 79), TCGA-CHOL (**E**; n = 36), and TCGA-LGG (**F**; n = 506). (**G**–**I**) The 1-, 3-, and 5-year ROC curves of the IRLPS for predicting progression-free survival in TCGA-ACC (**G**), TCGA-CHOL (**H**), and TCGA-LGG (I). (**J**–**L**) Kaplan–Meier survival curves of progression-free survival between patients with higher and lower risk scores of the IRLPS in TCGA-ACC (**J**), TCGA-CHOL (**K**), and TCGA-LGG (**L**).

To assess the robustness of the 18-IRLPS risk model, we test its predictive power on external cancer datasets with lincRNA expression and clinical information. Time-dependent ROC analysis revealed significant performances to predict overall survival for other types of cancer in the TCGA project, among which adrenocortical carcinoma (ACC), cholangiocarcinoma (CHOL), and low grade glioma (LGG) showed the highest AUC values (Fig. 7A–C and data not shown). Kaplan–Meier survival curves demonstrated that the 18-IRLPS could stratify patients from the TCGA-ACC, TCGA-CHOL, and TCGA-LGG datasets into high- and low-risk groups with significantly different overall survival using the same cutoff value of risk score (1.471) obtained from TCGA-LIHC (Fig. 7D–F). A similar extent of effectiveness was observed for progression-free survival of the three TCGA cancer types (Fig. 7G–L), confirming the powerful predictive performances of the IRLPS for overall survival and tumor progression.

Additionally, the role of the ten hub genes identified in TCGA-LIHC was explored in the three TCGA cancer types using Spearman's correlation and Cox regression analysis. The hub genes identified above were also significantly correlated with the IRLPS risk score in TCGA-ACC, TCGA-CHOL, and TCGA-LGG (r > 0.3; Supplementary Fig. 4A). They were also significantly associated with overall survival and progression-free survival (Supplementary Fig. 4B,C) in TCGA-ACC and TCGA-LGG. These findings demonstrate that the poor prognosis of IRLPS-high patients may be mediated by common underlying mechanisms in multiple cancer types.

## Discussion

HCC is highly heterogeneous both from molecular and clinical standpoints^28,29^, making insufficient responses to monotherapy in many clinical cases and the survival prediction quite complicated. Due to characteristics of the immune contexture substantially impacting immune therapy outcome^30^, the IRLPS was established from the tumor-specific immune-related lincRNAs as a prognostic indicator. Several recent reports screened lncRNAs associated with prognosis or immune response of HCC, using two to ten lncRNAs to construct a risk score formula^31–35^. Unlike traditional prognostic models, the pairwise comparison and score calculation of each IRLPs are based entirely on the lincRNA expression in the same patient, overcoming batch effects. Previous studies utilized a similar modeling strategy, of which 12 or 30 differentially expressed immune-related lncRNA pairs were included^36,37^. In contrast, we focused on only the intergenic subtype of lncRNA (lincRNA) and did not use arbitrary cutoffs for differential expression significance.

The 18-IRLPS consisted of 34 lincRNAs, some of which have been reported to be involved in the occurrence and development of liver cancer. For instance, the expression of DIO3OS is lower in HCC, and upregulation of DIO3OS represses malignant behaviors^38^. LINC01352 downregulation mediated by the HBx/ERα complex promotes HCC cell growth and metastasis^39^. Correspondingly, pairs of DIO3OS|RP11-672L10.6 and LINC01352|RP11-43F13.4 have an HR lower than 1 (Fig. 1B), indicating that higher expression of DIO3OS compared with RP11-672L10.6 or higher expression of LINC01352 compared with RP11-43F13.4 in a specific sample is associated with a better prognosis of patients with liver cancer.

The assessment of the immune status in the tumor microenvironment is essential for a comprehensive understanding of the tumor. A large number of studies have shown that dense infiltration of T cells, especially CD8^+^ T cells, predicts a good prognosis^22,40^. In most cancers, Th2 CD4^+^ T cells have been shown to support tumor growth and progression, which could form an immunosuppressive milieu to hamper the activation of CD8^+^ T cells for eradicating the tumor cells^41^. By integrating analysis, we revealed that the suppressive Th2 CD4^+^ T cells and M0 macrophages are more abundant in the IRLPS-high group, while the fraction of central memory CD8^+^ T cells decreased (Fig. 2F). Correlation analysis with immune-related gene sets also indicates that patients in the high-risk group were inversely associated with cytotoxic T cell, antigen presentation, and IFN signaling (Fig. 2E and Supplementary Fig. 3A). The infiltration of diverse types of immune cells is tightly regulated by various chemokines, which modulate tumor immunity, the biological phenotype of the tumors, and the prognosis for patients^42^. We found that the IRLPS-high group had decreased expression of CCL23 (Fig. 2H), in line with our previous study that CCL23 could serve as a tumor suppressor through recruiting CD8^+^ T cell infiltration in liver cancer^43^. Thus, the IRLPS may work through regulating immune cells infiltration and has a critical role in the immunosuppressive tumor microenvironment.

CD276, also known as B7-H3, is a newly identified target for cancer immunotherapy because it is overexpressed in tumor tissues while showing limited expression in most normal tissues^44^. To date, several CD276-based immunotherapeutic strategies have demonstrated potent antitumor activity and acceptable safety profiles in preclinical models^45^. In HCC, aberrantly expressed CD276 could promote tumor progression and inhibit the proliferation of CD8^+^ T cells^46^. Consistently, the expression level of CD276 was positively correlated with the IRLPS risk score and significantly associated with poor prognosis in overall survival. CD276 also had a positive correlation with Th2 CD4^+^ T cells and M0 macrophages (Fig. 3A–D), supporting the potential value of CD276 blockade in the IRLPS-based immunotherapy.

Several candidate compounds were identified to be potential treatments targeting the IRLPS in HCC patients. The dopamine receptor antagonists thioridazine and prochlorperazine are potential compounds targeting undifferentiated tumors^47^. A recent study shows that thioridazine is a potential drug against HCC through inhibition of the PI3K/AKT pathway, ROS induction, and angiogenesis^48^. Prochlorperazine possesses anticancer activity by affecting cell cycle stages, stimulating apoptosis, and inhibiting migration and invasiveness^49^. Cell proliferation inhibitor apigenin was also enriched in the high-risk group. Apigenin, a natural flavonoid, has low intrinsic toxicity and possesses preventive and therapeutic potential against cancers^50^. In HCC, apigenin could inhibit the growth and epithelial-mesenchymal transition of cancer cells^51,52^.

Aberrant expression of the identified hub genes may contribute to HCC occurrence and development. For instance, a recent study has demonstrated that CDCA8 promotes HCC growth and stemness through the AKT/β-catenin signaling^53^. BUB1B exerts an oncogenic effect in HCC cell proliferation, migration, and invasion partially by affecting mitochondrial function^54^. CDC20 could enhance cell proliferation and is associated with the development and progression of HCC^55^.

In summary, we established an 18-IRLPS, regardless of specific expression levels, as an independent prognostic indicator for patients with liver cancer. The risk model may also help distinguish immune and molecular characteristics and predict therapeutic sensitivity, highlighting the promising clinical significance for cancer patients’ personalized treatment and prognosis (Supplementary Fig. 5).

## Materials and methods

### Data collection and preprocessing

Transcriptome profiling (RNA-seq), clinical, and mutation data of the TCGA-LIHC project consisting of 50 normal and 374 tumor samples were obtained from the GDC Data Portal (https://portal.gdc.cancer.gov/). Patients with a follow-up time of 0 days were excluded. The GENCODE v22 GTF file for annotation and distinguish the mRNAs and lncRNAs was downloaded from https://gdc.cancer.gov/about-data/gdc-data-processing/gdc-reference-files. To determine tumor mutational burden (TMB), the total number of non-synonymous mutations was counted. The number of mutation events per million bases was calculated as the TMB for 361 samples. Tumor driver genes were identified by applying the maftool R package^56^.

Immune scores and Estimate scores, calculated by the ESTIMATE algorithm^57^, for samples within the TCGA-LIHC dataset were downloaded from https://bioinformatics.mdanderson.org/estimate/disease.html?liver%20hepatocellular%20carcinoma_RNAseqV2. Data of immune subtypes dominated in liver cancer, including C1 (wound healing), C2 (IFN-γ dominant), C3 (inflammatory), and C4 (lymphocyte depleted), were extracted from the supplementary table of a previous immunogenomic analysis ^25^.

### Immune infiltration analysis

To investigate the association between the IRLPS risk score and immune infiltrates in liver cancer, infiltration estimation for TCGA-LIHC was obtained from TIMER 2.0 (http://timer.comp-genomics.org/)^23^, which utilizes multiple immune deconvolution methods including TIMER^58^, XCELL^59^, QUANTISEQ^60^, MCPcounter^61^, EPIC^62^, CIBERSORT^63^, and CIBERSORT-ABS. The correlation (cor) values and *P*-values were acquired using Spearman's rank correlation test, with *P* < 0.05 as a significance threshold. The results were visualized in lollipop diagrams and scatter plots by the ggplot2 R package.

### Pairing immune-related lincRNAs

The relative expression levels of 429 tumor-specific immune-related lincRNA in each sample were pairwise compared as described previously^19,36^. Briefly, if the expression value of the first lincRNA is greater than the second in a specific sample, the score of this pair in this sample is 1; otherwise, it is 0. The score of each lincRNA pair was iteratively calculated for all samples. Some lincRNA pairs might be assigned to constant values (0 or 1 in most samples) because of biologically preferential transcription, making them unable to discriminate prognosis from one patient to another and non-informative. Therefore, lincRNA pairs with low variation (the score is 1 or 0 in more than 80% of the samples) were filtered^36^, and 35,604 valid lincRNA pairs were identified. After combining the survival data from the TCGA database, univariate Cox proportional hazards regression analysis was performed with *P* < 0.05 as the threshold, and 4288 overall survival-associated lincRNA pairs were extracted. Next, the least absolute shrinkage and selection operator (LASSO) regression was run 1000 times with the glmnet R package^64^ to prevent overfitting, and 39 lincRNA pairs were selected. Stepwise multivariate Cox regression analysis was conducted to further screen out an optimal combination from these pairs, and the 18 overall survival-associated signatures were obtained. *P* < 0.05 was set as the inclusion criteria.

The risk score for each patient was calculated based on the following formula: ${\text{risk}}\;{\text{score}} = \sum\nolimits_{i = 1}^{n} {\beta i*\lambda i}$, where n represents the numbers of lincRNA pairs included to construct the signature, β depicts the regression coefficient, and λ represents the 0-or-1 matrix of each lincRNA pair, respectively. We determined the optimal cutoff value based on a time-dependent receiver operating characteristic (ROC) curve to divide patients into high- or low-risk groups. The timeROC R package (version 0.4, weighting = ‘marginal’) was used to plot the time-dependent ROC curves and evaluate the values of area under the ROC curve (AUC)^65^.

### Small molecular compounds identification

To screen out candidate target compounds, differential expression analysis between the high- and low-risk groups was performed using the limma R package^66^ with the criteria of |log2(foldchange)| ≥  0.585 and FDR-adjusted *P* < 0.05. Differentially expressed genes (481 upregulated and 190 downregulated) were uploaded to the Connectivity Map web-server (https://portals.broadinstitute.org/cmap/).

### Chemotherapeutic response prediction

Chemosensitivity was predicted using the pRRophetic R package^67^ based on a public pharmacogenomics database, Genomics of Drug Sensitivity in Cancer. The half-maximal inhibitory concentration (IC50) on each sample was estimated by ridge regression. The prediction accuracy was assessed by tenfold cross-validation based on the training set.

### Gene set enrichment analysis

Gene set enrichment analysis (GSEA) was conducted between patients in different risk groups from the TCGA-LIHC cohort utilizing GSEA software (v4.1.0). We employed h.all.v7.4.symbols.gmt and c2.cp.kegg.v7.4.symbols.gmt as reference gene sets. The screening criteria of items were set as nominal *P* < 0.05 and FDR-adjusted q < 0.05.

### GO and KEGG enrichment analyses

To obtain the potential functions of the differentially expressed genes, GO and KEGG pathway enrichment analyses were implemented using the clusterProfiler and enrichplot R packages (*P* < 0.05, q < 0.05).

### Identification of hub genes

The STRING platform (https://www.string-db.org/) was employed to construct the protein–protein interaction (PPI) network using the differentially expressed genes with a confidence score > 0.9. The network was visualized using Cytoscape (v3.8.0). The cytoHubba plugin in Cytoscape was utilized to perform modular analysis, and the top ten hub genes were identified by the multi-network clustering algorithm.

### Statistical analyses

The statistical analyses were performed in R (v.4.1.0)^68^ with appropriate packages. The association of the 18-IRLPS or hub genes with patients' survival was examined by Cox hazards regression analysis using the survival R package^69^. The difference in survival between the high-risk and low-risk group of patients was determined using log-rank tests with the survival R package. The chi-square test was used for the categorical data, while the Wilcoxon and Kruskal tests were applied for two or more sets of continuous data.

### Ethics declarations

All methods were carried out in accordance with relevant guidelines and regulations.

## Supplementary Information

Supplementary Information.

## Data Availability

The data sets analysed during this study are available in public, open access repositories listed in this article.
