# Supplementary material for: Immune-related lincRNA pairs predict prognosis and therapeutic response in hepatocellular carcinoma
Source: Sci Rep. 2022 Mar 11;12:4259. doi: 10.1038/s41598-022-08225-w (PMC8917134; doi:10.1038/s41598-022-08225-w)
Supplement: Supplementary file 1 — Supplementary Information. [file 41598_2022_8225_MOESM1_ESM.docx]

**
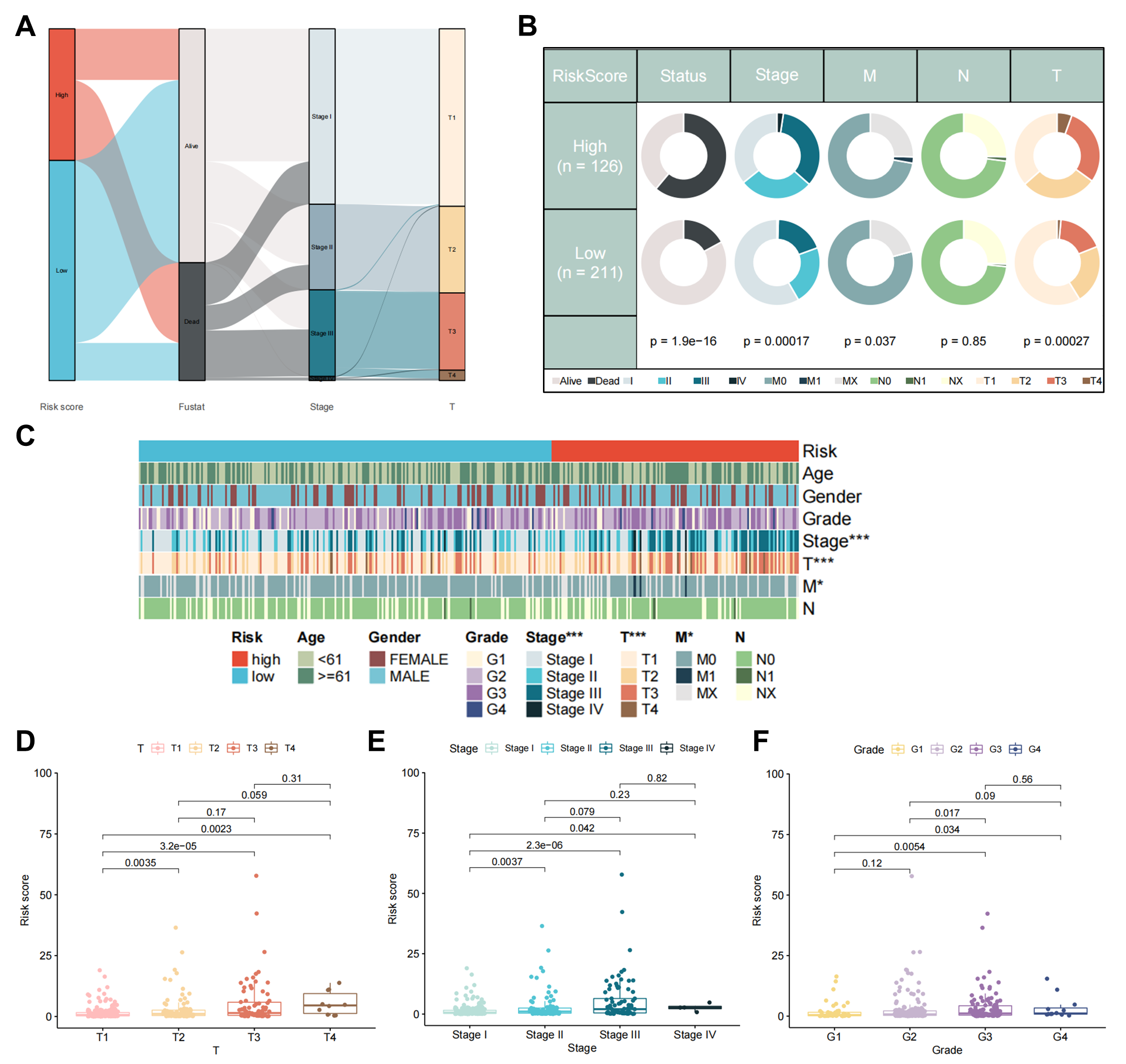
Supplementary Figure 1. The relationship between the IRLPS and clinicopathological characteristics.** (A) Alluvial diagram of patients distribution in groups with different risk, overall survival outcomes, clinical stage, and T stage. (B) Clinical divergence of clinical traits (survival status, clinical stage, M stage, N stage, and T stage) in the high-risk group and low-risk group using the chi-square test. (C-F) Strip chart (C) along with the scatter diagram showing that T stage (D), clinical stage (E), and tumor grade (F) were significantly associated with the risk score.

**
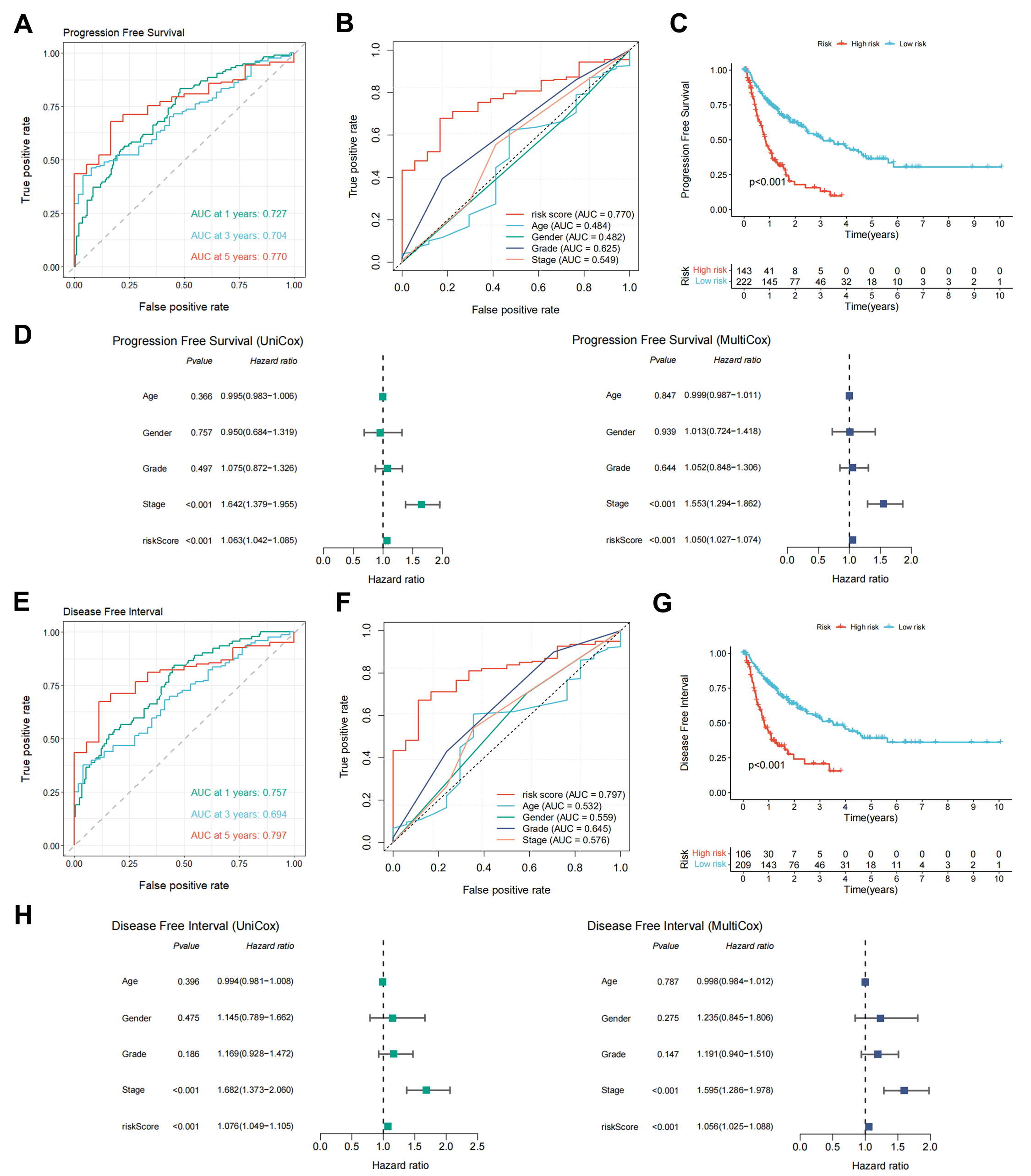
Supplementary Figure 2. The IRLPS is associated with tumor progression and recurrence.** (A) The 1-, 3-, and 5-year ROC curves of the IRLPS for predicting tumor progression. (B) Comparison of 5-year ROC of the IRLPS with that of other common clinical characteristics showed the superiority of the risk score. (C) Kaplan-Meier survival curves of progression-free survival between patients with higher and lower risk scores of the IRLPS. (D) Univariate (left) and multivariate (right) Cox hazard ratio analysis demonstrated that the risk score was an independent progression-free survival predictor. (E) The 1-, 3-, and 5-year ROC curves of the IRLPS for predicting tumor recurrence. (F) A comparison of 5-year ROC of the IRLPS with that of other common clinical characteristics. (G) Kaplan-Meier survival curves of disease-free interval between patients with higher and lower risk scores of the IRLPS. (H) Univariate (left) and multivariate (right) Cox hazard ratio analysis demonstrated that the risk score was an independent disease-free interval predictor.

**
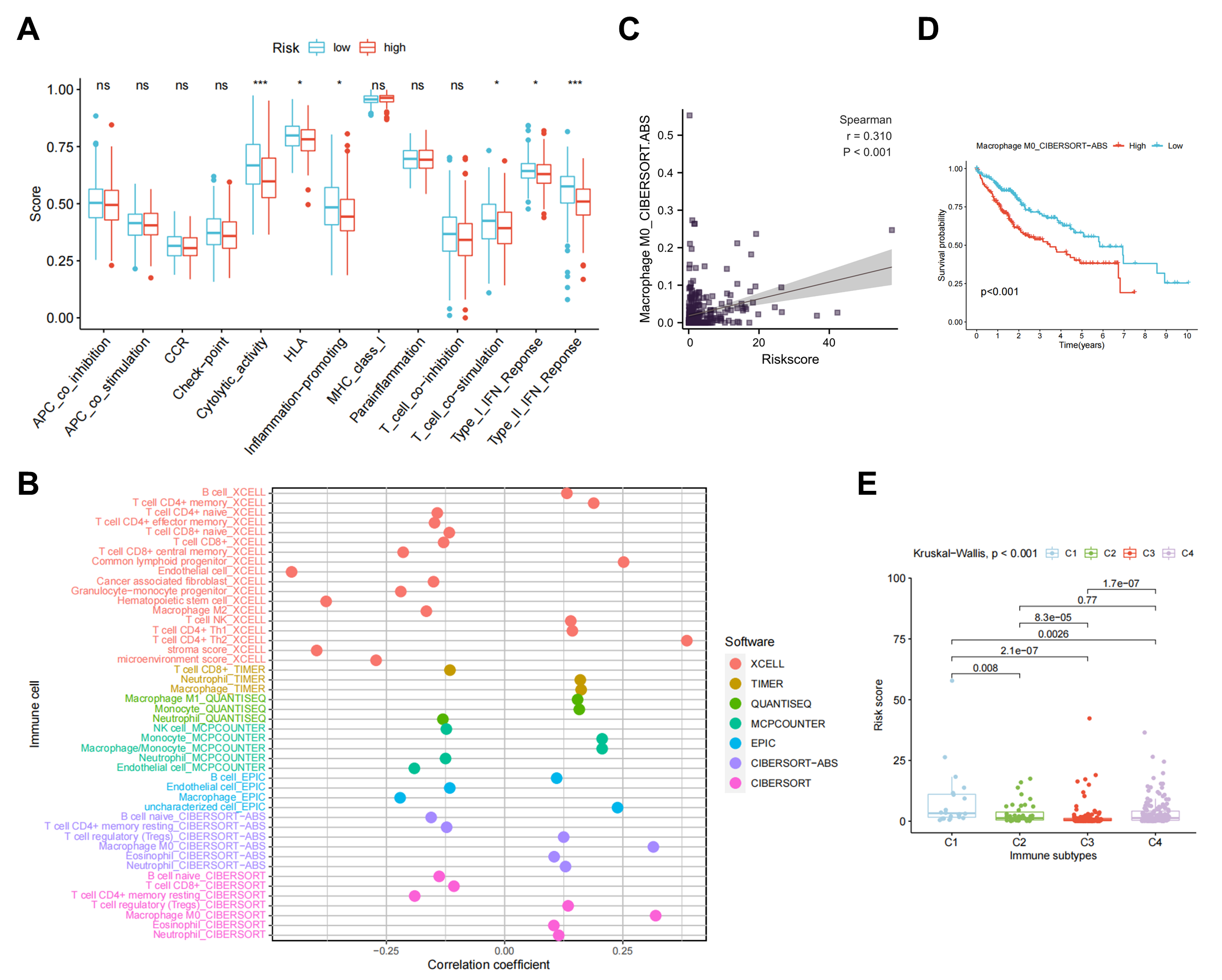
Supplementary Figure 3. Correlation between the prognostic risk score with immune cell infiltration.** (A) The enrichment scores of immune function-related gene sets in the two risk groups. (B) Lollipop diagram showing the correlation to tumor-infiltrating immune cell abundances obtained from TIMER2.0 by Spearman's rank test. (C) Association between the risk score of IRLPS and the absolute abundance of tumor-infiltrating M0 macrophages. (D) Kaplan-Meier survival curves of overall survival between patients with a higher and lower absolute abundance of tumor-infiltrating M0 macrophages. (E) Comparison of the risk score of IRLPS among four immune subtypes with the Kruskal test.

**
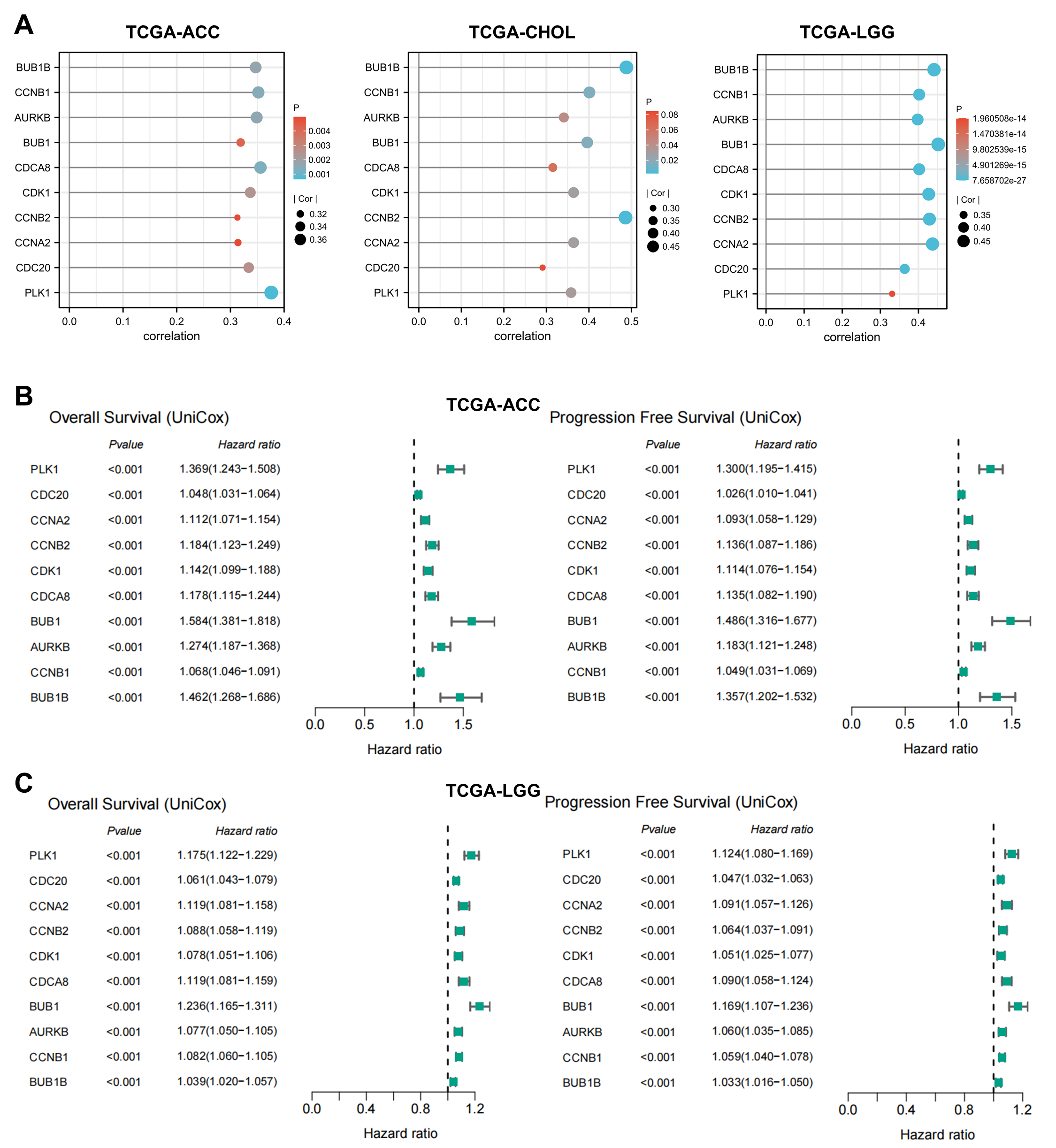
Supplementary Figure 4. Analysis of the hub genes identified from TCGA-LIHC in external datasets.** (A) Lollipop diagram showing the Spearman's correlation between the ten hub genes and the IRLPS risk score in TCGA-ACC, TCGA-CHOL, and TCGA-LGG. (B, C) Forest plot of univariate Cox regression analyses for overall survival and progression-free survival in TCGA-ACC (B) and TCGA-LGG (C).

**
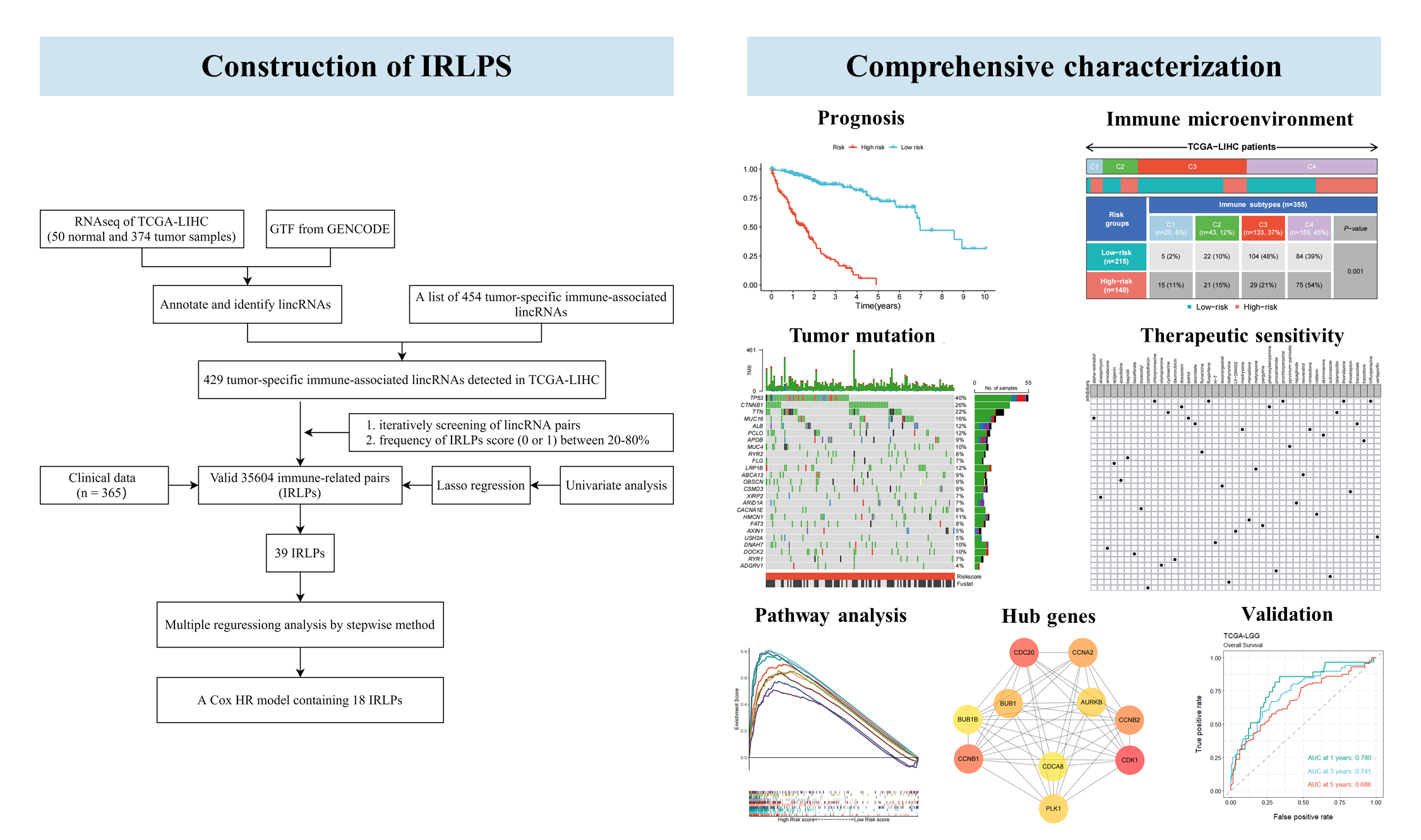
 Supplementary Figure 5. Graphical abstract for comprehensive characterization of the IRLPS.**

**Supplementary Table 1. Detailed information of 429 immune-related lincRNAs detected in TCGA-LIHC.**

| Symbol | Cluster | Tissue specificity |
| --- | --- | --- |
| AC004775.5 | C1 immune-inhibiting (cancer-shared) | tumor cell |
| AC005624.2 | C1 immune-inhibiting (cancer-shared) | tumor cell |
| AC007204.2 | C1 immune-inhibiting (cancer-shared) | tumor cell |
| AC024560.2 | C1 immune-inhibiting (cancer-shared) | tumor cell |
| AC069513.4 | C1 immune-inhibiting (cancer-shared) | tumor cell |
| AC073321.4 | C1 immune-inhibiting (cancer-shared) | tumor cell |
| AC079305.11 | C1 immune-inhibiting (cancer-shared) | tumor cell |
| AC093382.1 | C1 immune-inhibiting (cancer-shared) | tumor cell |
| AC133633.1 | C1 immune-inhibiting (cancer-shared) | tumor cell |
| AL928742.12 | C1 immune-inhibiting (cancer-shared) | tumor cell |
| AP000442.4 | C1 immune-inhibiting (cancer-shared) | tumor cell |
| AP000569.9 | C2 immune-inhibiting (cancer-specific) | tumor cell |
| AP001372.2 | C2 immune-inhibiting (cancer-specific) | tumor cell |
| AP001505.10 | C2 immune-inhibiting (cancer-specific) | tumor cell |
| AP001604.3 | C2 immune-inhibiting (cancer-specific) | tumor cell |
| AP006222.2 | C2 immune-inhibiting (cancer-specific) | tumor cell |
| BSN-AS2 | C2 immune-inhibiting (cancer-specific) | tumor cell |
| C12orf80 | C2 immune-inhibiting (cancer-specific) | tumor cell |
| C16orf47 | C2 immune-inhibiting (cancer-specific) | tumor cell |
| C21orf91-OT1 | C2 immune-inhibiting (cancer-specific) | tumor cell |
| C3orf79 | C2 immune-inhibiting (cancer-specific) | tumor cell |
| C6orf99 | C2 immune-inhibiting (cancer-specific) | tumor cell |
| CASC18 | C2 immune-inhibiting (cancer-specific) | tumor cell |
| CASC19 | C2 immune-inhibiting (cancer-specific) | tumor cell |
| CASC20 | C2 immune-inhibiting (cancer-specific) | tumor cell |
| CASC6 | C2 immune-inhibiting (cancer-specific) | tumor cell |
| CCDC26 | C2 immune-inhibiting (cancer-specific) | tumor cell |
| CH17-140K24.3 | C2 immune-inhibiting (cancer-specific) | tumor cell |
| CH17-437K3.1 | C2 immune-inhibiting (cancer-specific) | tumor cell |
| CH507-154B10.2 | C2 immune-inhibiting (cancer-specific) | tumor cell |
| CH507-24F1.2 | C2 immune-inhibiting (cancer-specific) | tumor cell |
| CH507-254M2.3 | C2 immune-inhibiting (cancer-specific) | tumor cell |
| CH507-42P11.6 | C2 immune-inhibiting (cancer-specific) | tumor cell |
| CH507-42P11.7 | C2 immune-inhibiting (cancer-specific) | tumor cell |
| CNTN4-AS2 | C2 immune-inhibiting (cancer-specific) | tumor cell |
| CTA-384D8.33 | C2 immune-inhibiting (cancer-specific) | tumor cell |
| CTA-992D9.7 | C2 immune-inhibiting (cancer-specific) | tumor cell |
| CTB-113P19.5 | C2 immune-inhibiting (cancer-specific) | tumor cell |
| CTB-129P6.11 | C2 immune-inhibiting (cancer-specific) | tumor cell |
| CTB-164N12.1 | C2 immune-inhibiting (cancer-specific) | tumor cell |
| CTB-32H22.1 | C2 immune-inhibiting (cancer-specific) | tumor cell |
| CTB-35F21.3 | C2 immune-inhibiting (cancer-specific) | tumor cell |
| CTB-41I6.2 | C2 immune-inhibiting (cancer-specific) | tumor cell |
| CTB-61M7.2 | C2 immune-inhibiting (cancer-specific) | tumor cell |
| CTC-235G5.3 | C2 immune-inhibiting (cancer-specific) | tumor cell |
| CTC-340D7.1 | C2 immune-inhibiting (cancer-specific) | tumor cell |
| CTC-429P9.2 | C2 immune-inhibiting (cancer-specific) | tumor cell |
| CTC-436K13.1 | C2 immune-inhibiting (cancer-specific) | tumor cell |
| CTC-436K13.6 | C2 immune-inhibiting (cancer-specific) | tumor cell |
| CTC-537E7.3 | C2 immune-inhibiting (cancer-specific) | tumor cell |
| CTC-542B22.1 | C2 immune-inhibiting (cancer-specific) | tumor cell |
| CTC-542B22.2 | C2 immune-inhibiting (cancer-specific) | tumor cell |
| CTC-543D15.3 | C2 immune-inhibiting (cancer-specific) | tumor cell |
| CTC-548K16.2 | C2 immune-inhibiting (cancer-specific) | tumor cell |
| CTD-2001C12.1 | C2 immune-inhibiting (cancer-specific) | tumor cell |
| CTD-2006K23.1 | C2 immune-inhibiting (cancer-specific) | tumor cell |
| CTD-2008A1.3 | C2 immune-inhibiting (cancer-specific) | tumor cell |
| CTD-2008L17.2 | C2 immune-inhibiting (cancer-specific) | tumor cell |
| CTD-2012J19.3 | C2 immune-inhibiting (cancer-specific) | tumor cell |
| CTD-2013N17.7 | C2 immune-inhibiting (cancer-specific) | tumor cell |
| CTD-2015G9.1 | C2 immune-inhibiting (cancer-specific) | tumor cell |
| CTD-2021H9.3 | C2 immune-inhibiting (cancer-specific) | tumor cell |
| CTD-2026J24.1 | C2 immune-inhibiting (cancer-specific) | tumor cell |
| CTD-2027I19.3 | C2 immune-inhibiting (cancer-specific) | tumor cell |
| CTD-2035E11.4 | C2 immune-inhibiting (cancer-specific) | tumor cell |
| CTD-2037L6.2 | C2 immune-inhibiting (cancer-specific) | tumor cell |
| CTD-2116N20.1 | C2 immune-inhibiting (cancer-specific) | tumor cell |
| CTD-2128A3.2 | C2 immune-inhibiting (cancer-specific) | tumor cell |
| CTD-2147F2.1 | C2 immune-inhibiting (cancer-specific) | tumor cell |
| CTD-2162K18.4 | C2 immune-inhibiting (cancer-specific) | tumor cell |
| CTD-2201E18.5 | C2 immune-inhibiting (cancer-specific) | tumor cell |
| CTD-2227I18.1 | C2 immune-inhibiting (cancer-specific) | tumor cell |
| CTD-2232E5.2 | C2 immune-inhibiting (cancer-specific) | tumor cell |
| CTD-2240J17.1 | C2 immune-inhibiting (cancer-specific) | tumor cell |
| CTD-2249K22.1 | C2 immune-inhibiting (cancer-specific) | tumor cell |
| CTD-2270L9.4 | C2 immune-inhibiting (cancer-specific) | tumor cell |
| CTD-2291D10.3 | C2 immune-inhibiting (cancer-specific) | tumor cell |
| CTD-2298J14.2 | C2 immune-inhibiting (cancer-specific) | tumor cell |
| CTD-2313J17.6 | C2 immune-inhibiting (cancer-specific) | tumor cell |
| CTD-2325M2.1 | C2 immune-inhibiting (cancer-specific) | tumor cell |
| CTD-2331D11.3 | C2 immune-inhibiting (cancer-specific) | tumor cell |
| CTD-2377D24.4 | C2 immune-inhibiting (cancer-specific) | tumor cell |
| CTD-2509G16.5 | C2 immune-inhibiting (cancer-specific) | tumor cell |
| CTD-2515H24.4 | C2 immune-inhibiting (cancer-specific) | tumor cell |
| CTD-2527I21.15 | C2 immune-inhibiting (cancer-specific) | tumor cell |
| CTD-2544H17.1 | C2 immune-inhibiting (cancer-specific) | tumor cell |
| CTD-2561B21.11 | C2 immune-inhibiting (cancer-specific) | tumor cell |
| CTD-2571L23.8 | C2 immune-inhibiting (cancer-specific) | tumor cell |
| CTD-2588E21.1 | C2 immune-inhibiting (cancer-specific) | tumor cell |
| CTD-2626G11.2 | C2 immune-inhibiting (cancer-specific) | tumor cell |
| CTD-2653M23.3 | C2 immune-inhibiting (cancer-specific) | tumor cell |
| CTD-3010D24.3 | C2 immune-inhibiting (cancer-specific) | tumor cell |
| CTD-3032H12.2 | C2 immune-inhibiting (cancer-specific) | tumor cell |
| CTD-3035D6.2 | C2 immune-inhibiting (cancer-specific) | tumor cell |
| CTD-3157E16.2 | C2 immune-inhibiting (cancer-specific) | tumor cell |
| CTD-3194G12.2 | C2 immune-inhibiting (cancer-specific) | tumor cell |
| CTD-3234P18.6 | C2 immune-inhibiting (cancer-specific) | tumor cell |
| DIO3OS | C2 immune-inhibiting (cancer-specific) | tumor cell |
| DSCR9 | C2 immune-inhibiting (cancer-specific) | tumor cell |
| ELFN1-AS1 | C2 immune-inhibiting (cancer-specific) | tumor cell |
| EPB41L4A-AS1 | C2 immune-inhibiting (cancer-specific) | tumor cell |
| EPHA5-AS1 | C2 immune-inhibiting (cancer-specific) | tumor cell |
| FAM157C | C2 immune-inhibiting (cancer-specific) | tumor cell |
| FAM225A | C2 immune-inhibiting (cancer-specific) | tumor cell |
| FAM225B | C2 immune-inhibiting (cancer-specific) | tumor cell |
| FAM66A | C2 immune-inhibiting (cancer-specific) | tumor cell |
| FAM87B | C2 immune-inhibiting (cancer-specific) | tumor cell |
| FLJ37505 | C2 immune-inhibiting (cancer-specific) | tumor cell |
| FLJ46066 | C2 immune-inhibiting (cancer-specific) | tumor cell |
| GS1-166A23.2 | C2 immune-inhibiting (cancer-specific) | tumor cell |
| GS1-304P7.3 | C2 immune-inhibiting (cancer-specific) | tumor cell |
| GS1-600G8.5 | C2 immune-inhibiting (cancer-specific) | tumor cell |
| HAR1B | C2 immune-inhibiting (cancer-specific) | tumor cell |
| JPX | C2 immune-inhibiting (cancer-specific) | tumor cell |
| KB-1410C5.3 | C2 immune-inhibiting (cancer-specific) | tumor cell |
| KB-1460A1.1 | C2 immune-inhibiting (cancer-specific) | tumor cell |
| KB-1466C5.1 | C2 immune-inhibiting (cancer-specific) | tumor cell |
| KB-1507C5.4 | C2 immune-inhibiting (cancer-specific) | tumor cell |
| KB-1592A4.14 | C2 immune-inhibiting (cancer-specific) | tumor cell |
| KB-7G2.9 | C2 immune-inhibiting (cancer-specific) | tumor cell |
| KC6 | C2 immune-inhibiting (cancer-specific) | tumor cell |
| LA16c-325D7.2 | C2 immune-inhibiting (cancer-specific) | tumor cell |
| LA16c-380A1.1 | C2 immune-inhibiting (cancer-specific) | tumor cell |
| LA16c-381G6.1 | C2 immune-inhibiting (cancer-specific) | tumor cell |
| LA16c-444G7.2 | C2 immune-inhibiting (cancer-specific) | tumor cell |
| LINC00028 | C2 immune-inhibiting (cancer-specific) | tumor cell |
| LINC00158 | C2 immune-inhibiting (cancer-specific) | tumor cell |
| LINC00221 | C2 immune-inhibiting (cancer-specific) | tumor cell |
| LINC00239 | C2 immune-inhibiting (cancer-specific) | tumor cell |
| LINC00284 | C2 immune-inhibiting (cancer-specific) | tumor cell |
| LINC00294 | C2 immune-inhibiting (cancer-specific) | tumor cell |
| LINC00322 | C2 immune-inhibiting (cancer-specific) | tumor cell |
| LINC00345 | C2 immune-inhibiting (cancer-specific) | tumor cell |
| LINC00364 | C2 immune-inhibiting (cancer-specific) | tumor cell |
| LINC00384 | C2 immune-inhibiting (cancer-specific) | tumor cell |
| LINC00396 | C2 immune-inhibiting (cancer-specific) | tumor cell |
| LINC00407 | C2 immune-inhibiting (cancer-specific) | tumor cell |
| LINC00421 | C2 immune-inhibiting (cancer-specific) | tumor cell |
| LINC00423 | C2 immune-inhibiting (cancer-specific) | tumor cell |
| LINC00426 | C2 immune-inhibiting (cancer-specific) | tumor cell |
| LINC00427 | C2 immune-inhibiting (cancer-specific) | tumor cell |
| LINC00434 | C2 immune-inhibiting (cancer-specific) | tumor cell |
| LINC00441 | C2 immune-inhibiting (cancer-specific) | tumor cell |
| LINC00443 | C2 immune-inhibiting (cancer-specific) | tumor cell |
| LINC00472 | C2 immune-inhibiting (cancer-specific) | tumor cell |
| LINC00473 | C2 immune-inhibiting (cancer-specific) | tumor cell |
| LINC00477 | C2 immune-inhibiting (cancer-specific) | tumor cell |
| LINC00479 | C2 immune-inhibiting (cancer-specific) | tumor cell |
| LINC00486 | C2 immune-inhibiting (cancer-specific) | tumor cell |
| LINC00526 | C2 immune-inhibiting (cancer-specific) | tumor cell |
| LINC00540 | C2 immune-inhibiting (cancer-specific) | tumor cell |
| LINC00563 | C2 immune-inhibiting (cancer-specific) | tumor cell |
| LINC00593 | C2 immune-inhibiting (cancer-specific) | tumor cell |
| LINC00601 | C2 immune-inhibiting (cancer-specific) | tumor cell |
| LINC00603 | C2 immune-inhibiting (cancer-specific) | tumor cell |
| LINC00637 | C2 immune-inhibiting (cancer-specific) | tumor cell |
| LINC00656 | C2 immune-inhibiting (cancer-specific) | tumor cell |
| LINC00670 | C2 immune-inhibiting (cancer-specific) | tumor cell |
| LINC00691 | C2 immune-inhibiting (cancer-specific) | tumor cell |
| LINC00861 | C2 immune-inhibiting (cancer-specific) | tumor cell |
| LINC00880 | C2 immune-inhibiting (cancer-specific) | tumor cell |
| LINC00900 | C2 immune-inhibiting (cancer-specific) | tumor cell |
| LINC00908 | C2 immune-inhibiting (cancer-specific) | tumor cell |
| LINC00910 | C2 immune-inhibiting (cancer-specific) | tumor cell |
| LINC00921 | C2 immune-inhibiting (cancer-specific) | tumor cell |
| LINC00923 | C2 immune-inhibiting (cancer-specific) | tumor cell |
| LINC00924 | C2 immune-inhibiting (cancer-specific) | tumor cell |
| LINC00934 | C2 immune-inhibiting (cancer-specific) | tumor cell |
| LINC00937 | C2 immune-inhibiting (cancer-specific) | tumor cell |
| LINC00964 | C2 immune-inhibiting (cancer-specific) | tumor cell |
| LINC00969 | C2 immune-inhibiting (cancer-specific) | tumor cell |
| LINC00977 | C2 immune-inhibiting (cancer-specific) | tumor cell |
| LINC01007 | C2 immune-inhibiting (cancer-specific) | tumor cell |
| LINC01028 | C2 immune-inhibiting (cancer-specific) | tumor cell |
| LINC01082 | C2 immune-inhibiting (cancer-specific) | tumor cell |
| LINC01087 | C2 immune-inhibiting (cancer-specific) | tumor cell |
| LINC01089 | C2 immune-inhibiting (cancer-specific) | tumor cell |
| LINC01091 | C2 immune-inhibiting (cancer-specific) | tumor cell |
| LINC01094 | C2 immune-inhibiting (cancer-specific) | tumor cell |
| LINC01108 | C2 immune-inhibiting (cancer-specific) | tumor cell |
| LINC01115 | C2 immune-inhibiting (cancer-specific) | tumor cell |
| LINC01122 | C2 immune-inhibiting (cancer-specific) | tumor cell |
| LINC01133 | C2 immune-inhibiting (cancer-specific) | tumor cell |
| LINC01150 | C2 immune-inhibiting (cancer-specific) | tumor cell |
| LINC01169 | C2 immune-inhibiting (cancer-specific) | tumor cell |
| LINC01179 | C2 immune-inhibiting (cancer-specific) | tumor cell |
| LINC01191 | C2 immune-inhibiting (cancer-specific) | tumor cell |
| LINC01197 | C2 immune-inhibiting (cancer-specific) | tumor cell |
| LINC01213 | C2 immune-inhibiting (cancer-specific) | tumor cell |
| LINC01227 | C2 immune-inhibiting (cancer-specific) | tumor cell |
| LINC01239 | C2 immune-inhibiting (cancer-specific) | tumor cell |
| LINC01258 | C2 immune-inhibiting (cancer-specific) | tumor cell |
| LINC01271 | C2 immune-inhibiting (cancer-specific) | tumor cell |
| LINC01272 | C2 immune-inhibiting (cancer-specific) | tumor cell |
| LINC01322 | C2 immune-inhibiting (cancer-specific) | tumor cell |
| LINC01348 | C2 immune-inhibiting (cancer-specific) | tumor cell |
| LINC01352 | C2 immune-inhibiting (cancer-specific) | tumor cell |
| LINC01355 | C2 immune-inhibiting (cancer-specific) | tumor cell |
| LINC01361 | C2 immune-inhibiting (cancer-specific) | tumor cell |
| LINC01366 | C2 immune-inhibiting (cancer-specific) | tumor cell |
| LINC01376 | C2 immune-inhibiting (cancer-specific) | tumor cell |
| LINC01411 | C2 immune-inhibiting (cancer-specific) | tumor cell |
| LINC01422 | C2 immune-inhibiting (cancer-specific) | tumor cell |
| LINC01444 | C2 immune-inhibiting (cancer-specific) | tumor cell |
| LINC01451 | C2 immune-inhibiting (cancer-specific) | tumor cell |
| LINC01475 | C2 immune-inhibiting (cancer-specific) | tumor cell |
| LINC01478 | C2 immune-inhibiting (cancer-specific) | tumor cell |
| LINC01479 | C2 immune-inhibiting (cancer-specific) | tumor cell |
| LINC01484 | C2 immune-inhibiting (cancer-specific) | tumor cell |
| LINC01502 | C2 immune-inhibiting (cancer-specific) | tumor cell |
| LINC01515 | C2 immune-inhibiting (cancer-specific) | tumor cell |
| LINC01516 | C2 immune-inhibiting (cancer-specific) | tumor cell |
| LINC01522 | C2 immune-inhibiting (cancer-specific) | tumor cell |
| LINC01543 | C2 immune-inhibiting (cancer-specific) | tumor cell |
| LINC01556 | C2 immune-inhibiting (cancer-specific) | tumor cell |
| LINC01558 | C2 immune-inhibiting (cancer-specific) | tumor cell |
| LINC01561 | C2 immune-inhibiting (cancer-specific) | tumor cell |
| LINC01563 | C2 immune-inhibiting (cancer-specific) | tumor cell |
| LINC01567 | C2 immune-inhibiting (cancer-specific) | tumor cell |
| LINC01572 | C2 immune-inhibiting (cancer-specific) | tumor cell |
| LL0XNC01-7P3.1 | C2 immune-inhibiting (cancer-specific) | tumor cell |
| LLNLR-246C6.1 | C2 immune-inhibiting (cancer-specific) | tumor cell |
| LUCAT1 | C2 immune-inhibiting (cancer-specific) | tumor cell |
| MGC45922 | C2 immune-inhibiting (cancer-specific) | tumor cell |
| MIR181A1HG | C2 immune-inhibiting (cancer-specific) | tumor cell |
| MIR22HG | C2 immune-inhibiting (cancer-specific) | tumor cell |
| PCAT19 | C2 immune-inhibiting (cancer-specific) | tumor cell |
| PKNOX2-AS1 | C2 immune-inhibiting (cancer-specific) | tumor cell |
| PTGES2-AS1 | C2 immune-inhibiting (cancer-specific) | tumor cell |
| RERG-AS1 | C2 immune-inhibiting (cancer-specific) | tumor cell |
| RP1-104O17.3 | C2 immune-inhibiting (cancer-specific) | tumor cell |
| RP1-140J1.1 | C2 immune-inhibiting (cancer-specific) | tumor cell |
| RP1-155D22.2 | C2 immune-inhibiting (cancer-specific) | tumor cell |
| RP1-15D23.2 | C2 immune-inhibiting (cancer-specific) | tumor cell |
| RP1-224A6.9 | C2 immune-inhibiting (cancer-specific) | tumor cell |
| RP1-228P16.5 | C2 immune-inhibiting (cancer-specific) | tumor cell |
| RP1-265C24.8 | C2 immune-inhibiting (cancer-specific) | tumor cell |
| RP1-269M15.3 | C2 immune-inhibiting (cancer-specific) | tumor cell |
| RP1-278O22.1 | C2 immune-inhibiting (cancer-specific) | tumor cell |
| RP1-28H20.3 | C2 immune-inhibiting (cancer-specific) | tumor cell |
| RP1-30E17.2 | C2 immune-inhibiting (cancer-specific) | tumor cell |
| RP1-56K13.5 | C2 immune-inhibiting (cancer-specific) | tumor cell |
| RP1-69D17.4 | C2 immune-inhibiting (cancer-specific) | tumor cell |
| RP11-1000B6.5 | C2 immune-inhibiting (cancer-specific) | tumor cell |
| RP11-1008C21.1 | C2 immune-inhibiting (cancer-specific) | tumor cell |
| RP11-1017G21.6 | C2 immune-inhibiting (cancer-specific) | tumor cell |
| RP11-1070N10.5 | C2 immune-inhibiting (cancer-specific) | tumor cell |
| RP11-1070N10.7 | C2 immune-inhibiting (cancer-specific) | tumor cell |
| RP11-107N15.1 | C2 immune-inhibiting (cancer-specific) | tumor cell |
| RP11-1080G15.2 | C2 immune-inhibiting (cancer-specific) | tumor cell |
| RP11-111A21.1 | C3 immune-promoting (cancer-shared) | tumor cell |
| RP11-111K18.2 | C3 immune-promoting (cancer-shared) | tumor cell |
| RP11-118K6.3 | C3 immune-promoting (cancer-shared) | tumor cell |
| RP11-142G1.3 | C3 immune-promoting (cancer-shared) | tumor cell |
| RP11-145H9.3 | C3 immune-promoting (cancer-shared) | tumor cell |
| RP11-200A1.1 | C3 immune-promoting (cancer-shared) | tumor cell |
| RP11-214O1.2 | C3 immune-promoting (cancer-shared) | tumor cell |
| RP11-245G13.2 | C3 immune-promoting (cancer-shared) | tumor cell |
| RP11-247A12.7 | C3 immune-promoting (cancer-shared) | tumor cell |
| RP11-255M2.3 | C3 immune-promoting (cancer-shared) | tumor cell |
| RP11-258C19.7 | C3 immune-promoting (cancer-shared) | tumor cell |
| RP11-269F21.3 | C3 immune-promoting (cancer-shared) | tumor cell |
| RP11-269G24.6 | C3 immune-promoting (cancer-shared) | tumor cell |
| RP11-277P12.9 | C3 immune-promoting (cancer-shared) | tumor cell |
| RP11-291L22.4 | C3 immune-promoting (cancer-shared) | tumor cell |
| RP11-2N1.3 | C3 immune-promoting (cancer-shared) | tumor cell |
| RP11-326N17.1 | C3 immune-promoting (cancer-shared) | tumor cell |
| RP11-329N22.1 | C3 immune-promoting (cancer-shared) | tumor cell |
| RP11-333A23.4 | C3 immune-promoting (cancer-shared) | tumor cell |
| RP11-342D11.3 | C3 immune-promoting (cancer-shared) | tumor cell |
| RP11-353K11.1 | C3 immune-promoting (cancer-shared) | tumor cell |
| RP11-359G22.2 | C3 immune-promoting (cancer-shared) | tumor cell |
| RP11-371M22.1 | C3 immune-promoting (cancer-shared) | tumor cell |
| RP11-387H17.4 | C4 immune-promoting (cancer-specific) | tumor cell |
| RP11-392O17.1 | C4 immune-promoting (cancer-specific) | tumor cell |
| RP11-394I13.1 | C4 immune-promoting (cancer-specific) | tumor cell |
| RP11-398A8.5 | C4 immune-promoting (cancer-specific) | tumor cell |
| RP11-398C13.6 | C4 immune-promoting (cancer-specific) | tumor cell |
| RP11-398E10.1 | C4 immune-promoting (cancer-specific) | tumor cell |
| RP11-398G24.2 | C4 immune-promoting (cancer-specific) | tumor cell |
| RP11-400N13.2 | C4 immune-promoting (cancer-specific) | tumor cell |
| RP11-403A21.1 | C4 immune-promoting (cancer-specific) | tumor cell |
| RP11-407A16.3 | C4 immune-promoting (cancer-specific) | tumor cell |
| RP11-407B7.3 | C4 immune-promoting (cancer-specific) | tumor cell |
| RP11-408A13.3 | C4 immune-promoting (cancer-specific) | tumor cell |
| RP11-410D17.2 | C4 immune-promoting (cancer-specific) | tumor cell |
| RP11-416I2.1 | C4 immune-promoting (cancer-specific) | tumor cell |
| RP11-41O4.2 | C4 immune-promoting (cancer-specific) | tumor cell |
| RP11-424D14.1 | C4 immune-promoting (cancer-specific) | tumor cell |
| RP11-426C22.4 | C4 immune-promoting (cancer-specific) | tumor cell |
| RP11-426C22.7 | C4 immune-promoting (cancer-specific) | tumor cell |
| RP11-42I10.1 | C4 immune-promoting (cancer-specific) | tumor cell |
| RP11-433J22.3 | C4 immune-promoting (cancer-specific) | tumor cell |
| RP11-437J19.1 | C4 immune-promoting (cancer-specific) | tumor cell |
| RP11-439A17.4 | C4 immune-promoting (cancer-specific) | tumor cell |
| RP11-43F13.4 | C4 immune-promoting (cancer-specific) | tumor cell |
| RP11-442J17.3 | C4 immune-promoting (cancer-specific) | tumor cell |
| RP11-445L6.3 | C4 immune-promoting (cancer-specific) | tumor cell |
| RP11-445P17.8 | C4 immune-promoting (cancer-specific) | tumor cell |
| RP11-445P19.3 | C4 immune-promoting (cancer-specific) | tumor cell |
| RP11-449P1.1 | C4 immune-promoting (cancer-specific) | tumor cell |
| RP11-44D19.1 | C4 immune-promoting (cancer-specific) | tumor cell |
| RP11-44F21.5 | C4 immune-promoting (cancer-specific) | tumor cell |
| RP11-460I19.2 | C4 immune-promoting (cancer-specific) | tumor cell |
| RP11-461O7.1 | C4 immune-promoting (cancer-specific) | tumor cell |
| RP11-465B22.8 | C4 immune-promoting (cancer-specific) | tumor cell |
| RP11-467I17.1 | C4 immune-promoting (cancer-specific) | tumor cell |
| RP11-468E2.5 | C4 immune-promoting (cancer-specific) | tumor cell |
| RP11-469J4.3 | C4 immune-promoting (cancer-specific) | tumor cell |
| RP11-473M20.16 | C4 immune-promoting (cancer-specific) | tumor cell |
| RP11-473O4.4 | C4 immune-promoting (cancer-specific) | tumor cell |
| RP11-474D1.3 | C4 immune-promoting (cancer-specific) | tumor cell |
| RP11-479J7.2 | C4 immune-promoting (cancer-specific) | tumor cell |
| RP11-486O13.4 | C4 immune-promoting (cancer-specific) | tumor cell |
| RP11-488C13.5 | C4 immune-promoting (cancer-specific) | tumor cell |
| RP11-488L18.10 | C4 immune-promoting (cancer-specific) | tumor cell |
| RP11-490B18.5 | C4 immune-promoting (cancer-specific) | tumor cell |
| RP11-493E12.1 | C4 immune-promoting (cancer-specific) | tumor cell |
| RP11-494O16.4 | C4 immune-promoting (cancer-specific) | tumor cell |
| RP11-498C9.15 | C4 immune-promoting (cancer-specific) | tumor cell |
| RP11-498P14.3 | C4 immune-promoting (cancer-specific) | tumor cell |
| RP11-502I4.3 | C4 immune-promoting (cancer-specific) | tumor cell |
| RP11-503C24.4 | C4 immune-promoting (cancer-specific) | tumor cell |
| RP11-509J21.2 | C4 immune-promoting (cancer-specific) | tumor cell |
| RP11-513N24.1 | C4 immune-promoting (cancer-specific) | tumor cell |
| RP11-514D23.2 | C4 immune-promoting (cancer-specific) | tumor cell |
| RP11-521D12.1 | C4 immune-promoting (cancer-specific) | tumor cell |
| RP11-522B15.3 | C4 immune-promoting (cancer-specific) | tumor cell |
| RP11-534L6.2 | C4 immune-promoting (cancer-specific) | tumor cell |
| RP11-539L10.2 | C4 immune-promoting (cancer-specific) | tumor cell |
| RP11-542M13.3 | C4 immune-promoting (cancer-specific) | tumor cell |
| RP11-543D5.1 | C4 immune-promoting (cancer-specific) | tumor cell |
| RP11-546K22.1 | C4 immune-promoting (cancer-specific) | tumor cell |
| RP11-54O7.16 | C4 immune-promoting (cancer-specific) | tumor cell |
| RP11-54O7.2 | C4 immune-promoting (cancer-specific) | tumor cell |
| RP11-554D14.6 | C4 immune-promoting (cancer-specific) | tumor cell |
| RP11-555G19.1 | C4 immune-promoting (cancer-specific) | tumor cell |
| RP11-561P12.5 | C4 immune-promoting (cancer-specific) | tumor cell |
| RP11-563J2.2 | C4 immune-promoting (cancer-specific) | tumor cell |
| RP11-567M16.2 | C4 immune-promoting (cancer-specific) | tumor cell |
| RP11-567M16.6 | C4 immune-promoting (cancer-specific) | tumor cell |
| RP11-567N4.3 | C4 immune-promoting (cancer-specific) | tumor cell |
| RP11-572M11.1 | C4 immune-promoting (cancer-specific) | tumor cell |
| RP11-575F12.3 | C4 immune-promoting (cancer-specific) | tumor cell |
| RP11-576D8.4 | C4 immune-promoting (cancer-specific) | tumor cell |
| RP11-599B13.3 | C4 immune-promoting (cancer-specific) | tumor cell |
| RP11-599B13.7 | C4 immune-promoting (cancer-specific) | tumor cell |
| RP11-60A24.3 | C4 immune-promoting (cancer-specific) | tumor cell |
| RP11-616M22.11 | C4 immune-promoting (cancer-specific) | tumor cell |
| RP11-61A14.4 | C4 immune-promoting (cancer-specific) | tumor cell |
| RP11-61O1.2 | C4 immune-promoting (cancer-specific) | tumor cell |
| RP11-642C5.1 | C4 immune-promoting (cancer-specific) | tumor cell |
| RP11-644L4.1 | C4 immune-promoting (cancer-specific) | tumor cell |
| RP11-66B24.2 | C4 immune-promoting (cancer-specific) | tumor cell |
| RP11-672L10.6 | C4 immune-promoting (cancer-specific) | tumor cell |
| RP11-679B19.2 | C4 immune-promoting (cancer-specific) | tumor cell |
| RP11-680H20.2 | C4 immune-promoting (cancer-specific) | tumor cell |
| RP11-686O6.2 | C4 immune-promoting (cancer-specific) | tumor cell |
| RP11-687M24.4 | C4 immune-promoting (cancer-specific) | tumor cell |
| RP11-688I9.4 | C4 immune-promoting (cancer-specific) | tumor cell |
| RP11-689C9.1 | C4 immune-promoting (cancer-specific) | tumor cell |
| RP11-6N13.1 | C4 immune-promoting (cancer-specific) | tumor cell |
| RP11-70D24.4 | C4 immune-promoting (cancer-specific) | tumor cell |
| RP11-713M6.2 | C4 immune-promoting (cancer-specific) | tumor cell |
| RP11-716H6.1 | C4 immune-promoting (cancer-specific) | tumor cell |
| RP11-718G2.5 | C4 immune-promoting (cancer-specific) | tumor cell |
| RP11-71E19.1 | C4 immune-promoting (cancer-specific) | tumor cell |
| RP11-734K21.5 | C4 immune-promoting (cancer-specific) | tumor cell |
| RP11-73G16.2 | C4 immune-promoting (cancer-specific) | tumor cell |
| RP11-74E22.6 | C4 immune-promoting (cancer-specific) | tumor cell |
| RP11-75L1.1 | C4 immune-promoting (cancer-specific) | tumor cell |
| RP11-761N21.1 | C4 immune-promoting (cancer-specific) | tumor cell |
| RP11-770G2.2 | C4 immune-promoting (cancer-specific) | tumor cell |
| RP11-778D9.13 | C4 immune-promoting (cancer-specific) | tumor cell |
| RP11-783K16.13 | C4 immune-promoting (cancer-specific) | tumor cell |
| RP11-797D24.3 | C4 immune-promoting (cancer-specific) | tumor cell |
| RP11-79H23.3 | C4 immune-promoting (cancer-specific) | tumor cell |
| RP11-810P12.5 | C4 immune-promoting (cancer-specific) | tumor cell |
| RP11-810P8.1 | C4 immune-promoting (cancer-specific) | tumor cell |
| RP11-819C21.1 | C4 immune-promoting (cancer-specific) | tumor cell |
| RP11-834C11.6 | C4 immune-promoting (cancer-specific) | tumor cell |
| RP11-849F2.9 | C4 immune-promoting (cancer-specific) | tumor cell |
| RP11-863P13.2 | C4 immune-promoting (cancer-specific) | tumor cell |
| RP11-888D10.3 | C4 immune-promoting (cancer-specific) | tumor cell |
| RP11-88H9.2 | C4 immune-promoting (cancer-specific) | tumor cell |
| RP11-88I21.2 | C4 immune-promoting (cancer-specific) | tumor cell |
| RP11-897M7.1 | C4 immune-promoting (cancer-specific) | tumor cell |
| RP11-89K21.1 | C4 immune-promoting (cancer-specific) | tumor cell |
| RP11-91J3.3 | C4 immune-promoting (cancer-specific) | tumor cell |
| RP11-932O9.10 | C4 immune-promoting (cancer-specific) | tumor cell |
| RP11-944C7.1 | C4 immune-promoting (cancer-specific) | tumor cell |
| RP13-20L14.1 | C4 immune-promoting (cancer-specific) | tumor cell |
| RP13-297E16.4 | C4 immune-promoting (cancer-specific) | tumor cell |
| RP13-463N16.6 | C4 immune-promoting (cancer-specific) | tumor cell |
| RP13-49I15.6 | C4 immune-promoting (cancer-specific) | tumor cell |
| RP13-507P19.2 | C4 immune-promoting (cancer-specific) | tumor cell |
| RP13-516M14.1 | C4 immune-promoting (cancer-specific) | tumor cell |
| RP13-714J12.1 | C4 immune-promoting (cancer-specific) | tumor cell |
| RP13-977J11.2 | C4 immune-promoting (cancer-specific) | tumor cell |
| RP4-550H1.5 | C4 immune-promoting (cancer-specific) | tumor cell |
| RP4-568C11.4 | C4 immune-promoting (cancer-specific) | tumor cell |
| RP4-614C15.2 | C4 immune-promoting (cancer-specific) | tumor cell |
| RP4-644L1.2 | C4 immune-promoting (cancer-specific) | tumor cell |
| RP4-712E4.2 | C4 immune-promoting (cancer-specific) | tumor cell |
| RP4-739H11.3 | C4 immune-promoting (cancer-specific) | tumor cell |
| RP4-758J18.10 | C4 immune-promoting (cancer-specific) | tumor cell |
| RP4-781K5.5 | C4 immune-promoting (cancer-specific) | tumor cell |
| RP4-781K5.6 | C4 immune-promoting (cancer-specific) | tumor cell |
| RP4-789D17.5 | C4 immune-promoting (cancer-specific) | tumor cell |
| RP5-1007H16.1 | C4 immune-promoting (cancer-specific) | tumor cell |
| RP5-1016A21.1 | C4 immune-promoting (cancer-specific) | tumor cell |
| RP5-1057I20.2 | C4 immune-promoting (cancer-specific) | tumor cell |
| RP5-1121E10.2 | C4 immune-promoting (cancer-specific) | tumor cell |
| RP5-1125A11.7 | C4 immune-promoting (cancer-specific) | tumor cell |
| RP5-1186P10.2 | C4 immune-promoting (cancer-specific) | tumor cell |
| RP5-856G1.2 | C4 immune-promoting (cancer-specific) | tumor cell |
| RP5-884M6.1 | C4 immune-promoting (cancer-specific) | tumor cell |
| RP5-899E9.1 | C4 immune-promoting (cancer-specific) | tumor cell |
| RP5-963E22.6 | C4 immune-promoting (cancer-specific) | tumor cell |
| RP6-109B7.4 | C4 immune-promoting (cancer-specific) | tumor cell |
| SCHLAP1 | C4 immune-promoting (cancer-specific) | tumor cell |
| SPATA3-AS1 | C4 immune-promoting (cancer-specific) | tumor cell |
| TTLL11-IT1 | C4 immune-promoting (cancer-specific) | tumor cell |
| UNQ6494 | C4 immune-promoting (cancer-specific) | tumor cell |
| WI2-2373I1.2 | C4 immune-promoting (cancer-specific) | tumor cell |
| WI2-87327B8.1 | C4 immune-promoting (cancer-specific) | tumor cell |
| WI2-87327B8.2 | C4 immune-promoting (cancer-specific) | tumor cell |
| XXbac-BPGBPG55C20.2 | C4 immune-promoting (cancer-specific) | tumor cell |
| ZNF582-AS1 | C4 immune-promoting (cancer-specific) | tumor cell |

**Supplementary Table 2. Association of the risk score with somatic variants.**

| Symbol | Higher risk score | Lower risk score | P value^#^ |
| --- | --- | --- | --- |
| TP53 | 55(40.15%) | 42(19.72%) | <0.0001 |
| DOCK2 | 14(10.22%) | 6(2.82%) | 0.0075 |
| DNAH7 | 14(10.22%) | 7(3.29%) | 0.0149 |
| HMCN1 | 15(10.95%) | 8(3.76%) | 0.0151 |
| LRP1B | 17(12.41%) | 11(5.16%) | 0.0253 |
| CSMD3 | 13(9.49%) | 12(5.63%) | 0.2484 |
| ADGRV1 | 5(3.65%) | 14(6.57%) | 0.3491 |
| OBSCN | 13(9.49%) | 14(6.57%) | 0.4279 |
| ALB | 17(12.41%) | 20(9.39%) | 0.4725 |
| FAT3 | 11(8.03%) | 12(5.63%) | 0.5081 |
| MUC4 | 14(10.22%) | 17(7.98%) | 0.5986 |
| PCLO | 16(11.68%) | 20(9.39%) | 0.6116 |
| AXIN1 | 7(5.11%) | 15(7.04%) | 0.6160 |
| CACNA1E | 11(8.03%) | 13(6.1%) | 0.6318 |
| MUC16 | 22(16.06%) | 29(13.62%) | 0.6333 |
| FLG | 9(6.57%) | 18(8.45%) | 0.6609 |
| ARID1A | 10(7.3%) | 12(5.63%) | 0.6885 |
| ABCA13 | 12(8.76%) | 15(7.04%) | 0.7022 |
| USH2A | 7(5.11%) | 14(6.57%) | 0.7399 |
| RYR1 | 9(6.57%) | 11(5.16%) | 0.7514 |
| TTN | 30(21.9%) | 51(23.94%) | 0.7542 |
| CTNNB1 | 36(26.28%) | 53(24.88%) | 0.8676 |
| XIRP2 | 9(6.57%) | 16(7.51%) | 0.9033 |
| APOB | 13(9.49%) | 20(9.39%) | >0.9999 |
| RYR2 | 11(8.03%) | 17(7.98%) | >0.9999 |

^#^Chi-square test.
